# CK2α-mediated phosphorylation of DUB3 promotes YAP1 stability and oncogenic functions

**DOI:** 10.1038/s41419-024-07323-z

**Published:** 2025-01-18

**Authors:** Lei Huang, Yalei Wen, Qin Guo, Caishi Zhang, Xiao Yang, Mei Li, YiXia Liu, Xinying Li, Jiaxin Tang, Xiaofeng Zhou, Qi Qi, Haoxing Zhang, Tongzheng Liu

**Affiliations:** 1 Department of General Surgery, Guangzhou Red Cross Hospital/State Key Laboratory of Bioactive Molecules and Druggability Assessment/International Cooperative Laboratory of Traditional Chinese Medicine Modernization and Innovative Drug Development of Ministry of Education (MOE) of China/College of Pharmacy, Jinan University, Guangzhou, China; 2 Research Institute for Maternal and Child Health, The Affiliated Guangdong Second Provincial General Hospital, Postdoctoral Research Station of Traditional Chinese Medicine, School of Pharmacy, Jinan University, Guangzhou, China; 3 Department of Pathology, Shanxi Provincial People’s Hospital, Taiyuan, China; 4 Jianli Traditional Chinese Medicine Hospital, Jingzhou, China; 5 School of Pharmacology, Lanzhou University, Lanzhou, China; 6 Guangdong Provincial Key Laboratory of Genome Stability and Disease Prevention, College of Life Sciences and Oceanography, Shenzhen University, Shenzhen, China; 7 State Key Laboratory of Bioactive Molecules and Druggability Assessment, MOE Key Laboratory of Tumor Molecular Biology, Guangdong Province Key Laboratory of Pharmacodynamic Constituents of TCM and New Drugs Research, Department of Pharmacology, School of Medicine, Jinan University, Guangzhou, China; 8 The State Key Laboratory of Functions and Applications of Medicinal Plants, Guizhou Medical University, Guiyang, China

**Keywords:** Oncogenes, Oncogenes

## Abstract

The aberrant upregulation of Yes-associated protein 1 (YAP1) in a variety of solid cancers contributes to tumor progression and poor clinical outcomes, rendering it an appealing therapeutic target. However, effective therapies to directly target YAP1 remain challenging. In this study, we perform a high-throughput screening and identify Casein kinase II (CK2) as an uncharacterized upstream regulator of YAP1 turnover in cancer cells of ovarian cancer and several other cancer types. Pharmacological inhibition of Casein kinase II by Silmitasertib or genetic depletion of the catalytic subunit of Casein kinase II (CK2α) markedly destabilizes YAP1 and consequently suppresses its oncogenic functions in vitro and in vivo. Moreover, we reveal that DUB3 as a bona fide deubiquitinase of YAP1, which functionally links CK2 and YAP1 stability in a variety of human cancers. Mechanistically, CK2α directly phosphorylates DUB3 at Thr495, thereby facilitating DUB3-mediated deubiquitination process of YAP1. On the contrary, the loss of Thr495 phosphorylation by the phosphorylation-defective mutant DUB3 T495A, the cancer-related mutant DUB3 D496H and CK2 inhibition failed to deubiquitinate and stabilize YAP1 effectively. Notably, upregulated expressions of CK2α and DUB3 in ovarian cancer positively correlate with YAP1 overexpression. Collectively, our findings demonstrate the functional significance of the CK2α-DUB3 axis in YAP1 stabilization and YAP1-driven tumor progression, highlighting that strategies to target this axis might be of benefit in the clinical management of ovarian cancer and several other lethal cancers with aberrantly upregulated YAP1.

## Introduction

Ovarian cancer is the deadliest gynecologic malignancy worldwide with limited effective therapies [1]. Cisplatin and other conventional chemotherapies remain standard treatments for ovarian cancer [2]. However, chemo-resistance frequently occurs and causes treatment failure in ovarian cancer [3]. Thus, identifying novel therapeutic targets and developing new strategies are urgently needed for the clinical management of ovarian cancer.

Yes-associated protein 1 (YAP1) is the key effector of the Hippo suppressive pathway which is pivotal in the regulation of tissue regeneration, organ development and cell proliferation [4, 5]. When the Hippo pathway is inhibited or the core kinase cascades MST/MAP4K/LATS are dysregulated, hypo-phosphorylated YAP1 translocated to the nucleus and interacts with transcriptional factors such as TEAD1-4 to induce target gene expressions, promoting cell proliferation, drug resistance, and metastasis [6]. Emerging evidence demonstrates that YAP1 upregulation of various solid cancer is positively correlated with tumor progression and poor survival outcomes [6–8]. For instance, increased YAP1 in tumors harboring BRAF V600E acts as a parallel survival factor and promote resistance to RAF and MEK inhibitor therapy [9]. YAP1 was reported to be highly upregulated in peritoneal carcinomatosis, conferring cancer stem cell properties and promoting metastasis [10, 11]. Additionally, several studies suggest that YAP1 may play a pivotal role in ovarian cancer, where elevated levels of YAP1 correlate with poor patient survival and serve as an independent prognostic marker [12, 13]. These findings underscore the potential of targeting YAP1 expression in ovarian cancer, and potentially other cancers, as a promising strategy for the development of novel therapeutic approaches.

Here, we revealed that the expression of YAP1 was significantly upregulated in ovarian cancer specimens compared to normal tissue. Furthermore, we demonstrated the therapeutic benefit of targeting YAP1 in ovarian cancer. Therefore, the identification of novel targets with actionable therapeutic drugs specially targeting YAP1 could greatly benefit the clinical outcome of ovarian cancer patients. Despite the importance of YAP1 in human cancers, the development of strategies to directly target YAP1 remains challenging [14]. For instance, Verteporfin, bis-aryl hydrazine scaffold and VGLL4 peptides were previously reported to inhibit the interaction between YAP1 and TEADs and display a tumor-suppressive effect in several cell and animal models [15]. However, the clinical potential of these compounds in human cancers is limited due to off-target effects and cytotoxicity [16]. Thus, targeting upstream regulators of YAP1 turnover might be an appealing strategy for the treatment of YAP1-driven cancers. Here, our high-throughput screening reveals that Silmitasertib, a clinically used Casein Kinase II inhibitor, leads to more than 90% decrease of YAP1 protein level and potently suppresses YAP1-driven ovarian cancer progression. More importantly, the tumor-suppressive activity of Casein kinase II inhibition on YAP1 stability and YAP1-dependent malignant processes were also observed in cancer cell lines of pancreatic ductal adenocarcinoma (PDAC), triple-negative breast cancer (TNBC), non-small cell lung cancer (NSCLC), colorectal cancer (CRC) and hepatocellular carcinoma (HCC). Mechanistically, Casein kinase II controls YAP1 stability through acting on DUB3, a previously unidentified deubiquitinating enzyme of YAP1. Furthermore, the upregulated expressions of CK2α and DUB3 positively correlate YAP1 expression in ovarian cancer specimens. Thus, our findings demonstrate the novel function of the CK2α-DUB3 axis in tumor progression by stabilizing YAP1 and highlight that targeting this axis might be an appealing strategy in human cancers with the aberrantly upregulated YAP1.

## Materials and methods

### Cell culture, plasmids and antibodies

Cancer cell lines A2780, SKOV3, HEK293T, HCT-116, LoVo, MIAPaCa-2, PANC-1, H460, A549, Huh-7, MDA-MB-231 and 786-O were purchased from the American Type Culture Collection. RCC4 was kindly provided by Dr Zhenkun Lou’s lab. Cell lines were authenticated by short tandem repeat DNA profiling analysis and maintained mycoplasma-free. A2780, SKOV3, PANC-1, MIAPaCa-2, MDA-MB-231, RCC4, Huh-7 and HEK293T cells were cultured in DMEM medium with 10% FBS (ExCell Bio). HCT-116 cells were cultured in McCoy’s 5a Medium Modified with 10% FBS. LoVo, A549, 786-O and H460 cells were cultured in RPMI-1640 medium with 10% FBS.

YAP1, Casein kinase II alpha (CK2α) and DUB3 were cloned into pGEX4T-1 (containing GST tag), pET30a (containing His tag), pIRES (containing FLAG and S tag), pLV.3 (containing FLAG tag), pLV.6 (containing GFP tag) and pLV.5 (containing HA and S tag). Site-directed mutagenesis (TOYOBO) was utilized to generate site mutations. YAP1, CK2α and DUB3 shRNAs were from Sigma-Aldrich Co. YAP1 shRNA sequences are 5′-CAGGTGATACTATCAACCAAA-3′ and 5′-GCCACCAAGCTAGATAAAGAA-3′; CK2α shRNA sequences are 5′- CGTAAACAACACAGACTTCAA -3′ and 5′- CGATTATAGTTTGGATATGTG-3′; DUB3 shRNA sequences are 5′- GGGAAATACCTGCTACGAGAA -3′ and 5′- GCACCTTAGACCACTGGAAAT -3′.

Antibodies against YAP1 (66900-1-Ig), TAZ (23306-1-AP), MYC (60003-2-Ig), DUB3 (26143-1-AP) and CK2α (10992-1-AP) were purchased from Proteintech Group Co. Anti-FLAG (F1804), anti-HA (H3663) anti-*β*-actin (A1978) antibodies and anti-Flag affinity gel were purchased from Sigma-Aldrich Co. Anti-ubiquitin (sc-8017) antibody was purchased from Santa Cruz Biotechnology. Anti-CK2 substrate-specific antibody (8738S) was purchased from Cell Signaling Technology. Light chain or heavy chain specific IPKine™ HRP were purchased from Abbkine Scientific Co.Ltd. Anti-HA protein agaroses and Anti-Myc Magnetic Beads were purchased from Beyotime Biotechnology.

### Screening of small molecular compounds

The in-house small molecule compounds library contains 1139 compounds supplied in 96-well plates. A2780 cells were infected with lentiviruses encoding GFP-tagged YAP1 and treated with vehicle (DMSO) or compounds at final concentration of 10 μM for 24 h (n = 3). The intensity of green fluorescence was determined at 24 h after treatment of vehicle or compounds. The intensity of green fluorescence was measured by a high-throughput multi-mode micropore detection system and quantified in relation to the vehicle.

### Immunoprecipitation assay

For endogenous interaction, cell lysates of A2780 or SKOV3 cells were incubated with protein A/G beads and indicated antibodies for 4 h at 4 °C. After four times washing, immunoprecipitates were boiled in 50 μL loading buffer and subjected to immunoblotting. For the ubiquitination assay and other experiments, indicated constructs were transfected into cells. HA-protein agaroses or anti-Flag affinity gel were used to immunoprecipitate target proteins from cell lysates. After four times of bead washing, immunoprecipitates were subject to immunoblotting.

### Glutathione S-transferase (GST) pull-down assay

Indicated pGEX4T-1 and pET30a constructs were transformed in Escherichia coli strain BL21. After the induction by IPTG, GST or GST-fusion proteins were pulled down by Pierce Glutathione agarose and incubated with purified His-tagged protein at 4 °C for 2 h. After four times of bead washing, the in vitro interaction was measured by immunoblotting.

### Quantitative real-time PCR (qRT-PCR)

TRIzol reagent (Thermo Scientific, MA, USA) was utilized to extract RNA from cultured cells. FastKing gDNA Dispelling RT SuperMix (Tiangen, Beijing, China) was utilized to reverse transcribed RNA to cDNA. FastFire qPCR PreMix (SYBR Green) were used for qRT-CR. All PCR assays were done in triplicate with GAPDH as internal control. Relative levels of target genes were standardized to GAPDH mRNA level and normalized to control group. Primer sequences are as follows. *GAPDH* Forward: ATGGGGAAGGTGAAGGTCG, Reverse: GGGGTCATTGATGGCAACAATA, *YAP1* Forward: TGTCCCAGATGAACG TCACAGC, Reverse: TGGTGGCTGTTTCACTGGAGCA, *CYR61* Forward: GGAAAAGGCAGCTCACTGAAGC, Reverse: GGAGATACCAGTTCCACAGGTC, *ANKRD1* Forward: CGACTCCTGATTATGTATGGCGC, Reverse: GCTTTGGTT CCATTCTGCCAGTG, *CTGF* Forward: CCAATGACAACGCCTCCTG, Reverse: GAGCTTTCTGGCTGCACCA.

### Denaturating Ni-NTA pull-down assay

Indicated cells were transfected with constructs. Cell pellets were lysed in denaturating buffer containing 8 M urea, 300 mM NaCl, 0.1 M NaH_2_PO_4_, and 0.01 M Tris (pH 8.0). Cell lysates were sonicated and mixed with Ni-NTA agarose beads (Invitrogen) at 4 °C for 4 h. After four times washing, Ni-NTA agarose beads were incubated with 50 μL loading buffer and boiled. SDS–polyacrylamide gel electrophoresis and immunoblotting were performed to examine the ubiquitination level of indicated proteins.

### In vitro ubiquitination assay

Indicated constructs were transfected in cells followed by the treatment of MG-132 (10 μM) for 10 h. HA protein agarose was utilized to immunoprecipitate YAP1 in cell lysate. Indicated pGEX4T-1 constructs were transformed in Escherichia coli strain BL21. After the induction by IPTG, Glutathione agarose was used to pulldown recombinant GST-fusion DUB3 WT and C89S mutant followed by elusion with GSH buffer containing 10 mM GSH, 50 mM Tris HCl, pH 8.0, and concentration by ultrafiltration. Immunoprecipitated YAP1 was incubated with GST or GST-fusion proteins at 4 °C for 4 h and polyubiquitylated YAP1 was measured by western blot.

### In vitro kinase assay

Vector or HA-CK2α constructs were transfected into HEK293T cells. Following a 48 h period, anti-HA protein agaroses were used to immunoprecipitate CK2α in cell lysate. Recombinant GST-fused DUB3 WT and T495A proteins were purified with Pierce glutathione agarose and then eluted. CK2α immunoprecipitates were next mixed with purified GST, GST-DUB3 WT or T495A at 30 °C for 30 min in the kinase buffer containing 50 mM Tris-HCl, pH 7.4, 10 mM *β*-glycerophosphate, 50 mM NaCl, 10 mM MgCl_2_, 1 mM dithiothreitol and 100 μM ATP. Bacterially purified GST-CK2α and GST-CK2α K68M protein was incubated with purified His-DUB3 or His-DUB3 T495A (200 ng) proteins at 30 °C for 30 min in the kinase buffer. The reaction was terminated by adding SDS–PAGE loading buffer and heating the mixture to 100 °C for 5 min. The samples were then analyzed by SDS–PAGE [17].

### Cell proliferation assay

A2780, SKOV3, MDA-MB-231, MIAPaCa-2 (2 × 10^4^ per well) or H460 cells (4 × 10^4^ per well) were seeded. At the indicated time, cells were trypsinized by using 0.25% trypsin and centrifuged. Cell pellets were washed and re-suspended in PBS before counting under the microscope.

### CCK8 assay

In a 96-well plate, 2000 cells per well were seeded. Cell viability was assessed with the CCK8 Assay (MCE) by measuring optical density values at 450 nm wavelength.

### Immunohistochemical staining

Sections of ovarian cancer tissues were provided by the tissue bank of The First Affiliated Hospital of Jinan University under the approval document of the Institutional Medical Ethics Committee (Ethics Approval License: JNUKY-2023-0062). IHC assays were conducted on paraffin-embedded specimens of ovarian cancer patients as described previously [18] using anti-CK2α (10992-1-AP), anti-DUB3 (ab129931) and YAP1 (66900-1-Ig). Two pathologists rated the intensity of immunostaining blindly. The statistic correlation between YAP1 and DUB3 in ovarian cancer tissues was conducted using the χ2-test and Pearson’s correlation coefficient.

### Animal studies

For subcutaneous xenografting, A2780 cells (1 × 10^6^ cells per mouse) were subcutaneously implanted into Female BALB/c nude mice (n = 6). Female BALB/c nude mice (5–6 weeks old) were provided by Yaokang Biotechnology Co, Ltd, Jiangsu, China and were housed under specific-pathogen-free condition in the Animal Center of Jinan University. Animal sample sizes and experimental settings were determined according to our previous publication [19]. When tumors grew to 100-150 mm^3^, mice were randomly grouped and administered with saline, Silmitasertib (60 mg/kg, p.o. twice daily), cisplatin (5 mg/kg i.p. every three days) or Silmitasertib plus cisplatin [20, 21]. After mice were scarified, tumors were surgically removed and weighed. Animal experiments were performed under the approval document of Jinan University Institutional Animal Care and Use Committee (20230406-02).

### Statistics

Cell proliferation and survival experiments was conducted independently at least in triplicate, with results represented as the mean ± S.D. Statistic significance was determined using the following p-values: *P < 0.05; **P < 0.01; ***P < 0.001; ****P < 0.0001. Animal study data were represented as mean ± S.D. of six mice. Statistical analysis was performed using GraphPad Prism software (Version 9.3). All data were compared by Tukey’s test, t-test, one-way ANOVA analysis or two-way ANOVA analysis.

## Results

### Casein kinase II is a novel regulator of YAP1 turnover in multiple types of cancers

Ovarian cancer is the deadliest gynecologic malignancy without effective therapeutic strategies. Although the aberrant upregulation of YAP1 in several cancer types was demonstrated to promote tumor progression [22, 23], the status and regulatory mechanisms of YAP1 in ovarian cancer are not fully understood. We first analyzed YAP1 expression in ovarian cancer and revealed the significantly upregulated YAP1 expression in ovarian cancer tissues compared to normal tissues (Fig. S1A). The depletion of YAP1 in A2780 and SKOV3 cells strongly suppressed cell proliferation (Fig. S1B–D) and sensitized ovarian cancer cells to cisplatin (Fig. S1E and F), while the ectopically overexpressed of YAP1 displayed opposite effects (Fig. S1G–K). These results indicate that targeting YAP1 might benefit the management of ovarian cancer.

**Fig. 1:**
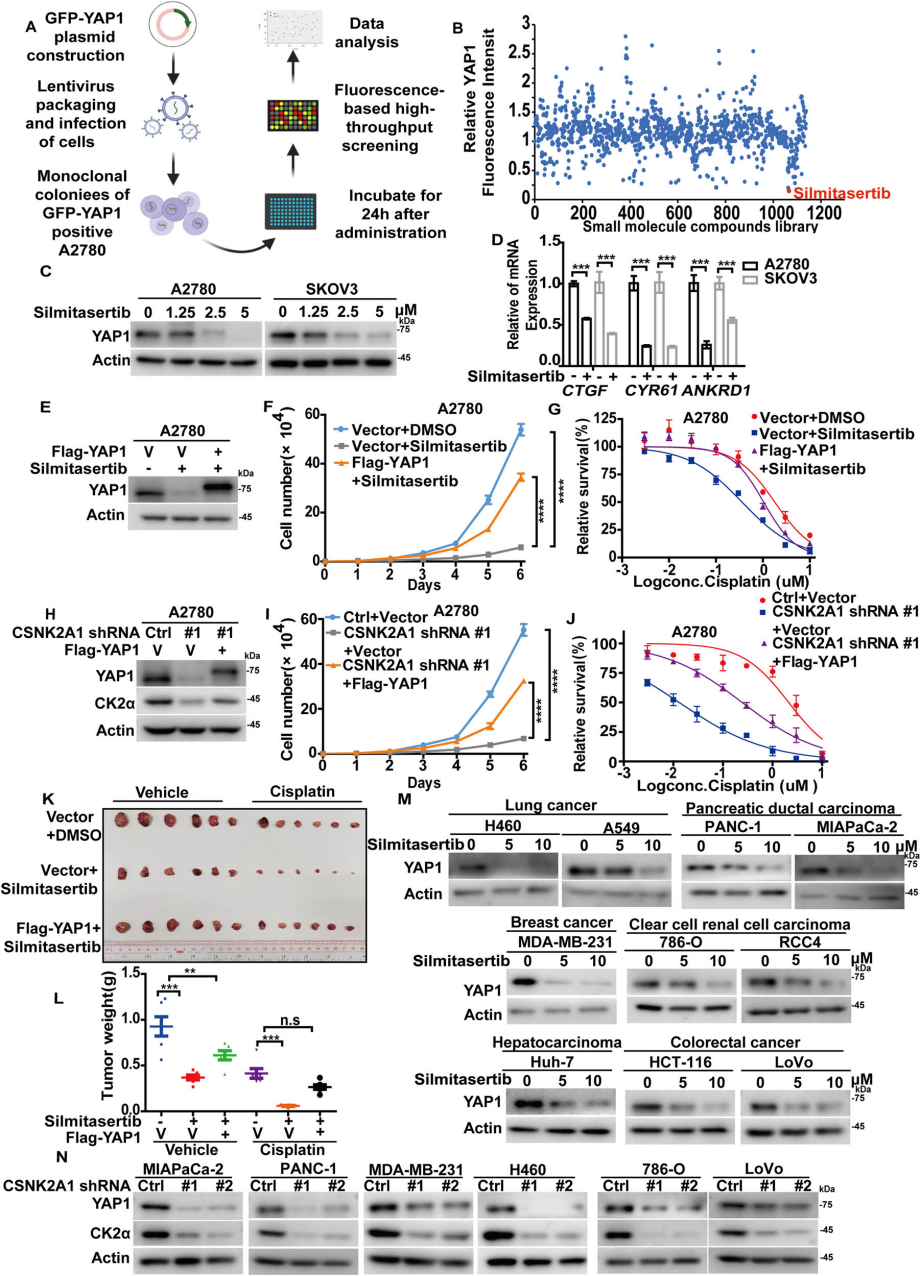
Casein kinase II is a novel regulator of YAP1 turnover in multiple types of cancers. **A** Strategy used to identify small molecule compounds mitigating GFP-YAP1 stability. **B** Among screening hits with in-house small molecule compounds library, the specific inhibitor of Casein kinase II, Silmitasertib, caused the highest inhibitory effect on YAP1. **C** A2780 and SKOV3 cells were treated with Silmitasertib at different concentrations, and the protein level of YAP1 was measured by immunobloting. **D** A2780 and SKOV3 cells were treated with DMSO or Silmitasertib. mRNA levels of YAP1 target genes *CTGF*, *CYR61* and *ANKRD1* were measured by qRT-PCR (n = 3), standardized to GAPDH and normalized. **E** Vector (V) or Flag-YAP1 were transfected into A2780 cells and then treated with DMSO or Silmitasertib. The protein level of YAP1 was measured by immunoblotting. **F** The proliferation of cells used in (**E**) was measured and analyzed. **G** Indicated concentrations of Cisplatin were treated into cells used in (**E**), and a CCK8 assay was performed to measure cell survival. **H** Vector (V) or Flag-YAP1 were transfected in Control (Ctrl) or CK2α-depleted A2780 cells, and the protein level of YAP1 was measured by immunoblotting. **I** The proliferation of cells used in (**H**) was measured and analyzed. **J** Indicated concentrations of Cisplatin were treated into cells used in (**H**), and a CCK8 assay was performed to measure cell survival. **K** and **L** Cells generated in (**E**) were implanted subcutaneously into nude mice (n = 6). When tumors grew to around 100–150 mm^3^, mice were randomly grouped and administered with saline, Silmitasertib (60 mg/kg, p.o. twice per day), cisplatin (5 mg/kg i.p. every 3 days), or a combination of Silmitasertib and cisplatin. Tumors were collected (**K**) and tumor weights were analyzed (**L**). **M** Silmitasertib was treated in H460, A549, PANC-1, MIAPaCa-2, MDA-MB-231, 786-O, RCC4, Huh-7, HCT-116 and LoVo cells for 24 h. The protein level of YAP1 was measured by immunoblotting. **N** MIAPaCa-2, PANC-1, MDA-MB-231, H460, 786-O and LoVo cells with the depletion of CK2α were generated. YAP1 and CK2α protein levels were measured by immunoblotting.

Considering the lack of effective strategies directly targeting deregulated YAP1 in human cancers, we next screened for small molecule compounds to mitigate YAP1 stability by determining the fluorescence intensity in A2780 cells stably expressing GFP-YAP1 (Fig. 1A). As shown in Fig. 1B, the fluorescence intensity of GFP-YAP1 was dramatically reduced by several compounds, among which Silmitasertib, a selective inhibitor of CK2 granted orphan drug designation by the FDA for cholangiocarcinoma and currently assessed in several other trials [24, 25], caused the strongest inhibitory effect. We next investigated the role of CK2 on YAP1 turnover and function in ovarian cancer. As shown in Fig. 1C-D, the enzymatic inhibition of CK2 by Silmitasertib in A2780 and SKOV3 cells dramatically decreased the protein level of YAP1, which resulted in the downregulated mRNA levels of YAP1 target genes *CTGF*, *CYR61* and *ANKRD1* [26]. Consistently, depletion of alpha catalytic subunit of CK2 (CK2α) significantly reduced the endogenous YAP1 level in A2780 cells (Fig.1E, Fig. 1H). Intriguingly, the reduced levels of overexpressed Flag-YAP1 in A2780 cells following CK2α depletion or inhibition by silmitasertib were also observed (Fig. S2A, B). Next, we found that treatment of Silmitasertib or CK2α depletion could significantly inhibit cell proliferation of A2780 and SKOV3 and increase cellular sensitivity to cisplatin (Fig.1E-J, Fig. S2C-H), which could be largely rescued by the reconstitution of YAP1. In line with these in vitro results, similar results were obtained in an in vivo animal model (Fig. 1K-L). Taken together, these results revealed that targeting CK2 significantly suppress ovarian cancer in a YAP1-dependent manner.

Given the frequent upregulation of YAP1 and CK2α in a variety of human cancers, we next investigated whether CK2α regulated YAP1 in other cancer types. As shown in Fig. 1M, N, the regulation of YAP1 by CK2 might be a common mechanism as the treatment of Silmitasertib or the depletion of CK2α significantly reduced YAP1 protein levels in various cancer cells including lung cancer (H460 and A549), PDAC (PANC-1 and MIAPaCa-2), TNBC (MDA-MB-231), renal cancer (786-O and RCC4), HCC (Huh-7) and CRC (HCT-116 and LoVo). Consistently, the treatment of Silmitasertib or the depletion of CK2α in H460, MIAPaCa-2 and MDA-MB-231 cells significantly inhibited cell proliferation and increased cellular sensitivity to cisplatin or Gemcitabine, which could be markedly rescued by the reconstitution of YAP1 (Fig. S2I–N). These results highlight the clinical potentials of targeting CK2 in the management of various types of human cancer by destabilizing YAP1.

### CK2 stabilizes YAP1 by affecting its ubiquitination

**Fig. 2:**
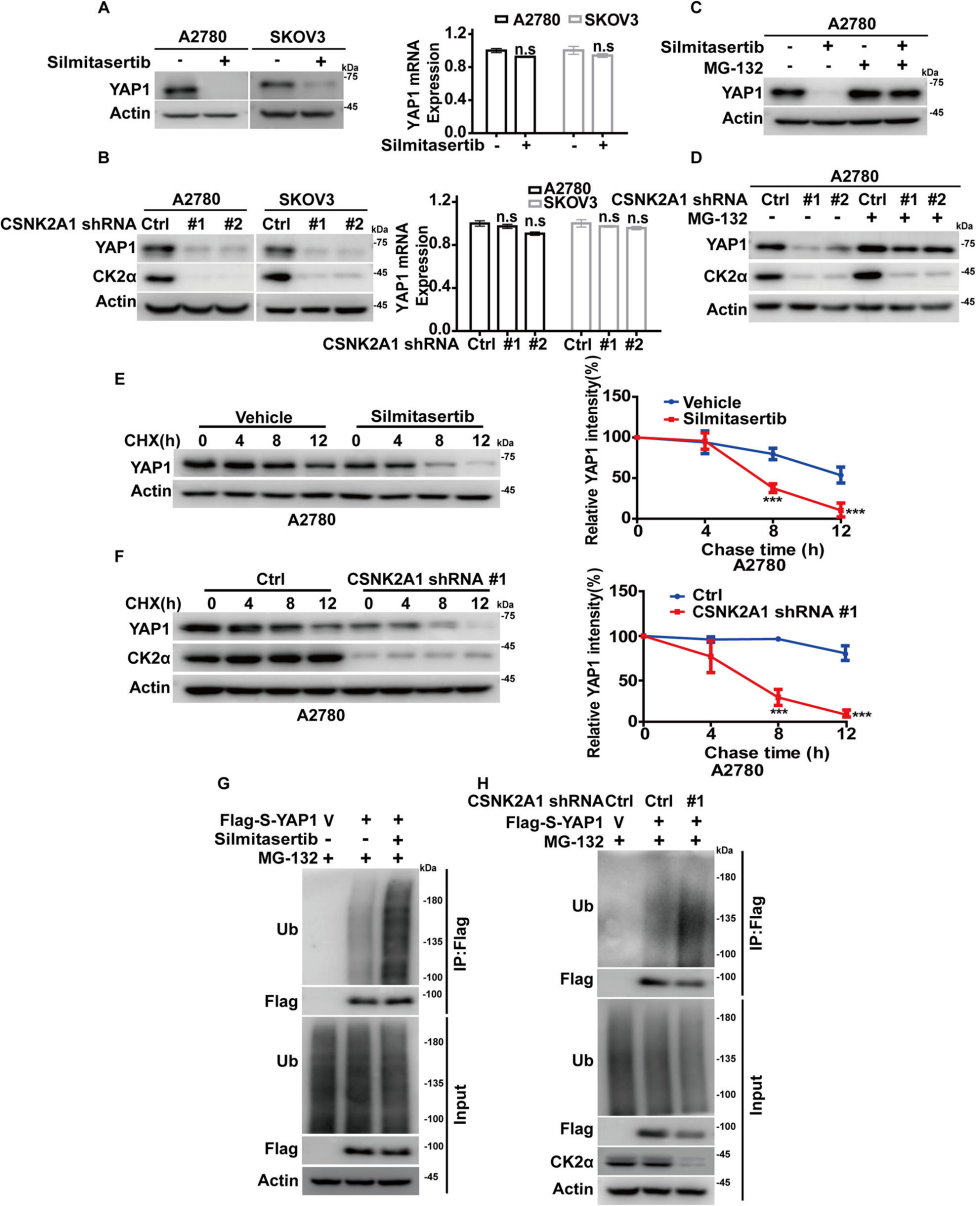
CK2α stabilizes YAP1 by affecting its ubiquitination. **A** A2780 and SKOV3 cells were treated with Silmitasertib, and the protein level of YAP1 was measured by immunoblotting. YAP1 mRNA levels were measured by qRT-PCR (n = 3), standardized to GAPDH and normalized. **B** CK2α-depleted A2780 and SKOV3 cells were generated. Protein levels of YAP1 and CK2α were measured by immunoblotting. YAP1 mRNA levels were measured by qRT-PCR (n = 3). **C** A2780 cells were treated with Silmitasertib followed by treatment of either DMSO or MG-132 (10 μM) for 10 h. The protein level YAP1 was measured by immunoblotting. **D** CK2α-depleted A2780 cells were subjected to DMSO or MG-132 (10 μM) treatment. Protein levels of YAP1 and CK2α were measured by immunoblotting. **E** A2780 cells were pretreated with Silmitasertib and treated with cycloheximide (200 μg/mL). Cell lysate was collected at the indicated times, and YAP1 protein levels were measured by immunoblotting. The level of YAP1 relative to Actin was analyzed by image J. **F** Control (Ctrl) or CK2α-depleted A2780 cells were treated with cycloheximide (200 μg/mL). Cell lysates were collected at the indicated times, and the protein levels of YAP1 were measured by immunoblotting. The relative level of YAP1 to Actin was analyzed by image J. **G** Indicated constructs were transfected into cells, and cells were pretreated with vehicle or Silmitasertib followed by 10 h exposure of MG-132 (10 μM). Anti-Flag affinity gel was used to immunoprecipitate YAP1 in cell lysates, and the ubiquitination level of YAP1 was measured by immunoblotting. **H** Vector (V) or Flag-S-YAP1 were transfected into control (Ctrl) or CK2α-depleted cells followed by 10 h of exposure to MG-132 (10 μM). Anti-Flag affinity gel was used to immunoprecipitate YAP1 in cell lysates. The ubiquitination level of YAP1 was measured by immunoblotting.

We next investigated underlying mechanisms by which CK2 regulates YAP1. As shown in Fig. 2A, B, the inhibition of CK2 by Silmitasertib or the depletion of CK2α in A2780 cells and SKOV3 cells significantly reduced YAP1 protein levels without affecting its mRNA levels. Intriguingly, the proteasome inhibitor MG-132 restored the decreased YAP1 protein level in CK2α-deficient cells or in Silmitasertib-treated cells, suggesting a proteasome-dependent mechanism in the regulation of YAP1 by CK2 (Fig. 2C, D, Fig. S3A, B). In addition, results of cycloheximide pulse-chase assay showed that YAP1 half-life was much shorter in CK2α-depleted cells or Silmitasertib-treated cells (Fig. 2E, F, Fig. S3C, D), which might be due to the increased level of YAP1 ubiquitination (Fig. 2G, H). TAZ (Transcriptional coactivator with PDZ-binding motif), a homolog of YAP1, shares many functional and structural similarities with YAP1. Given this, it is plausible that CK2α may regulate TAZ turnover in a manner similar to YAP1. As showed in Fig. S3E, F, treatment with Silmitasertib or CK2α depletion in A2780 cells resulted in a significant reduction in TAZ protein levels. This decrease in TAZ expression may be attributed to altered ubiquitination of TAZ (Fig. S3G, H). These findings indicate that CK2 stabilizes YAP1 and TAZ by modulating its ubiquitination, thus preventing its proteasome-dependent degradation.

### DUB3 is a bona fide deubiquitinase of YAP1

**Fig. 3:**
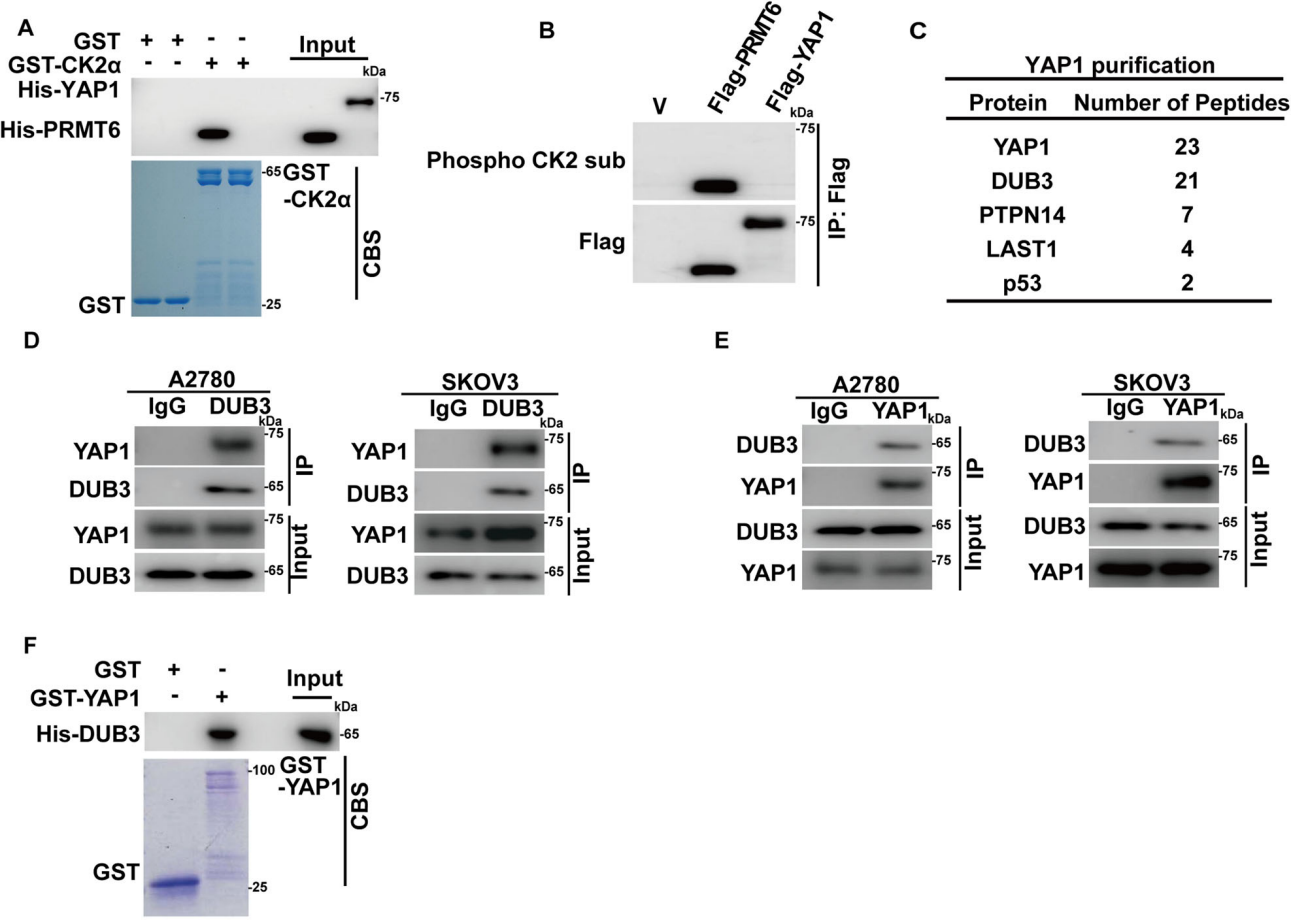
DUB3 is a bona fide YAP1 deubiquitinase. **A** The direct interactions between bacterially purified GST-CK2α and His-YAP1 or His-PRMT6 were assessed using an in vitro interaction assay. Coomassie blue staining (CBS) was performed to quantify levels of GST and GST-CK2α. **B** A2780 cells were transfected with either vector (V), Flag-PRMT6 or Flag-YAP1. Anti-Flag affinity gel was used to immunoprecipitate Flag-YAP1 or Flag-PRMT6. Subsequently, a phospho-CK2 substrate antibody was used to assess the phosphorylation levels of YAP1 and PRMT6. **C** Flag-YAP1 was transfected into A2780 cells. Mass spectrometry analysis of YAP1-immunoprecipitates was used to identify potential YAP1 interactors. Cell lysates of A2780 and SKOV3 cells were immunoprecipitated using IgG, anti-DUB3 (**D**) or anti-YAP1 (**E**) antibodies. The endogenous interaction of YAP1-DUB3 was measured by immunoblotting. **F** The interaction of bacterially purified GST-YAP1and His-DUB3 was measured by an in vitro interaction assay. CBS was performed to examine GST and GST-YAP1 levels.

Given that CK2 phosphorylates and regulates the degradation of various proteins, such as BMI1, BRMS1 and other protein substrates [27, 28], we speculate that the CK2 might directly phosphorylate and regulate YAP1 turnover. However, CK2α directly interacts with PRMT6 and effective phosphorylates it, PRMT6 as a positive control, as it is a well-established substrate for CK2α [29]. Conversely, we did not detect a direct interaction between bacterial purified GST-CK2α and His-YAP1 in vitro, nor did we observe phosphorylation of YAP1 using a CK2α substrate-specific antibody (Fig. 3A-B). These surprising results strongly suggest that CK2 could not act on YAP1 directly and an unidentified intermediary factor might be essential to mediate the regulation of YAP1 by CK2.

Previous studies have demonstrated that the phosphorylation of YAP1 regulates its subcellular localization and stability. Specifically, phosphorylation at Ser 127 by LATS1/2 kinases promotes YAP1 binding to 14-3-3 proteins, sequestering YAP1 in the cytoplasm, while phosphorylation at Ser397 promotes recruitment of the SCF (beta-TRCP) E3 ubiquitin ligase complex, leading to ubiquitinates YAP1 ubiquitination and subsequent proteasome-dependent degradation [30, 31]. To explore whether CK2 influences the phosphorylation of YAP1 at these sites, we assessed the effects of CK2 inhibition or depletion on p-S127 and p-S397 levels. As shown in Fig. S3I-J, treatment with Silmitasertib or CK2α depletion in the presence of MG-132 did not significantly affect the phosphorylation of YAP1 at these sites. We further explored whether CK2 modulates YAP1 stability through its interaction with β-TRCP. However, neither treatment with Silmitasertib nor CK2α depletion affected the interaction between YAP1 and β-TrCP, indicating that CK2α does not regulate YAP1 stability through β-TRCP-mediated degradation (Fig. S3K-L). To identify the potential linker between CK2 and YAP1, tandem affinity purification and mass spectrometry analysis were performed within A2780 cells expressing Flag-YAP1. As shown in Fig. 3C, apart from several reported YAP1-interacting proteins such as PTPN14 and LAST1 [32–34], the deubiquitinase DUB3 was identified as one potential YAP1 interactor. Further, we confirmed endogenous interaction of YAP1 and DUB3 in A2780 and SKOV3 cells (Fig. 3D, E,). In addition, direct interaction between purified GST-YAP1 and His-DUB3 was also detected (Fig. 3F), suggesting that DUB3 could directly interact with YAP1.

**Fig. 4:**
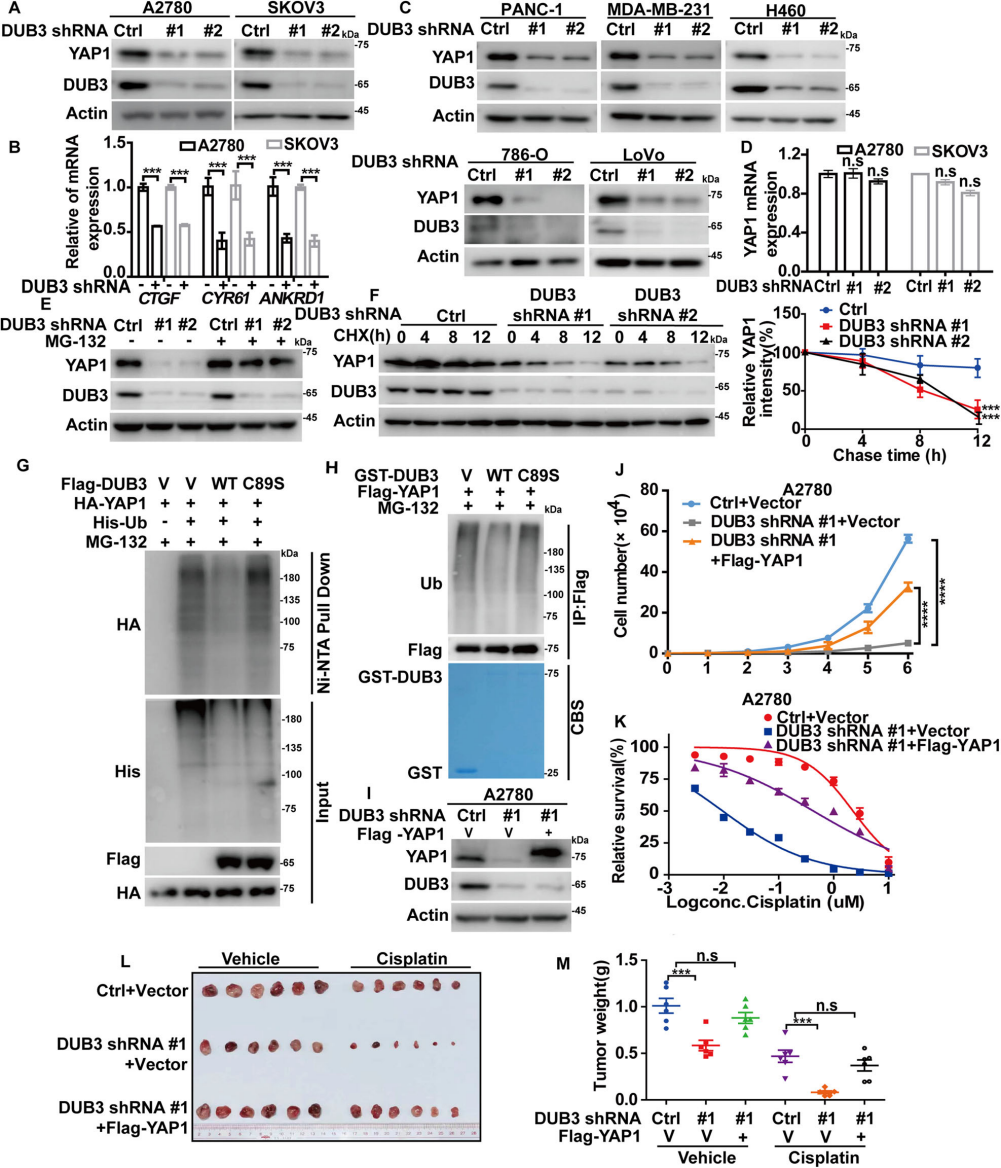
DUB3 stabilizes YAP1 and promotes tumor progression. **A** Control (Ctrl) or DUB3-depleted A2780 and SKOV3 cells were generated. Immunoblotting was performed to examine protein levels of YAP1 and DUB3. **B** mRNA levels of YAP1 target genes CTGF, CYR61, and ANKRD1 were measured in Control (Ctrl) or CK2α-depleted A2780 and SKOV3 cells by qRT-PCR (n = 3). **C** Protein levels of YAP1 and DUB3 were measured in control (Ctrl) and DUB3-depleted PANC-1, MDA-MB-231, H460, 786-O and LoVo cells by immunoblotting. **D** mRNA levels of YAP1 were measured in Control (Ctrl) or DUB3-depleted A2780 and SKOV3 cells by qRT-PCR (n = 3). **E** Control (Ctrl) or DUB3-depleted A2780 cells were subjected to vehicle or MG-132 (10 μM) for 10 h. YAP1 and DUB3 levels were measured by immunoblotting. **F** Control (Ctrl) or DUB3-depleted A2780 cells were treated with cycloheximide (200 μg/mL). Cell lysates were collected at the indicated times, and YAP1 protein levels were measured by immunoblotting (n = 3). The relative level of YAP1 to Actin was analyzed by image J. **G** Indicated constructs were transfected into cells, and His-tagged ubiquitin was pull-downed by Ni-NTA beads. The ubiquitination level of YAP1 was examined by immunoblotting. **H** The indicated constructs were transfected into cells, and MG-132 (10 μM) was administered for 10 h. Flag-YAP1 was immunoprecipitated using anti-Flag affinity gel and then was mixed with recombinant GST, GST-DUB3 or GST-DUB3 C89S. The polyubiquitination of YAP1 was examined by immunoblotting. A Coomassie Brilliant Blue (CBS) staining was performed to assess the levels of GST or GST-fusion proteins. **I** Vector (V) or Flag-YAP1 were transfected in Control (Ctrl) or DUB3-depleted A2780 cells, and the level of YAP1 was examined by immunoblotting. **J** The proliferation of cells generated in (**I**) was measured and analyzed. **K** Indicated concentrations of Cisplatin were treated into cells generated in (**I**), and a CCK8 assay was used to measure cell survival. **L, M** Cells in (**I**) (1 × 10^6^ per mice) were subcutaneously implanted into nude mice (n = 6). When tumors grew to around 150 mm^3^, mice were randomly grouped and administered with saline or cisplatin (5 mg/kg i.p. every 3 days). Tumor weights were measured (**L**) and analyzed (**M**).

We next investigated whether DUB3 could regulate YAP1 stability. The depletion of DUB3 in A2780 and SKOV3 cells significantly decreased the protein level of endogenous YAP1 and the transcription of YAP1 target genes (Fig. 4A-B). As shown in Fig. S4A, the depletion of DUB3 also resulted in a significant reduction of overexpressed Flag-YAP1 levels. Intriguingly, the knockdown of DUB3 in PANC-1, MDA-MB-231, H460, 786-O and LoVo cells dramatically reduced the protein level of YAP1 as well (Fig. 4C), implying that DUB3 might be a pan-cancerous regulator of YAP1. Moreover, we discovered that the knockdown of DUB3 in ovarian cancer cells did not affect the transcription of YAP1 (Fig. 4D), while the proteasome inhibition by MG-132 restored the YAP1 protein level caused by DUB3-depletion (Fig. 4E, Fig. S4B). In addition, the half-life of YAP1 protein was much shorter in DUB3-depleted cells (Fig. 4F, Fig. S4C). These results indicate that DUB3 regulates the YAP1 level through ubiquitin-proteasome pathway. Consistently, we performed ubiquitination assays in different conditions and found that DUB3 WT rather than the inactive mutant C89S strongly reduced the ubiquitination level of YAP1 (Fig. 4G). Furthermore, incubation with purified GST-DUB3 WT but not the inactive mutant C89S significantly reduced polyubiquitinated YAP1 levels in vitro (Fig. 4H). We also found that DUB3 depletion in A2780 cells significantly reduced TAZ protein levels (Fig. S4D). Moreover, effect of DUB3 on TAZ protein levels is enzyme activity-dependent, as WT DUB3, but not the inactive C89S mutant, efficiently reduced TAZ ubiquitination levels (Fig. S4E).

We next investigated whether DUB3 could promote ovarian cancer progression in YAP1-dependent manner. As shown in Fig. 4I-M and Fig. S4F-H, DUB3 depletion in A2780 and SKOV3 cells significantly decreased cell proliferation and sensitized cancer cells to cisplatin in vitro and in vivo, whereas reconstitution of YAP1 in DUB3-depleted cells could markedly rescued these phenotypical changes. These findings clearly indicate the novel tumor-promoting role of DUB3 in ovarian cancer as well as several other cancer types at least in part by controlling YAP1 stability.

### CK2α interacts with and phosphorylates DUB3 at Thr495

**Fig. 5:**
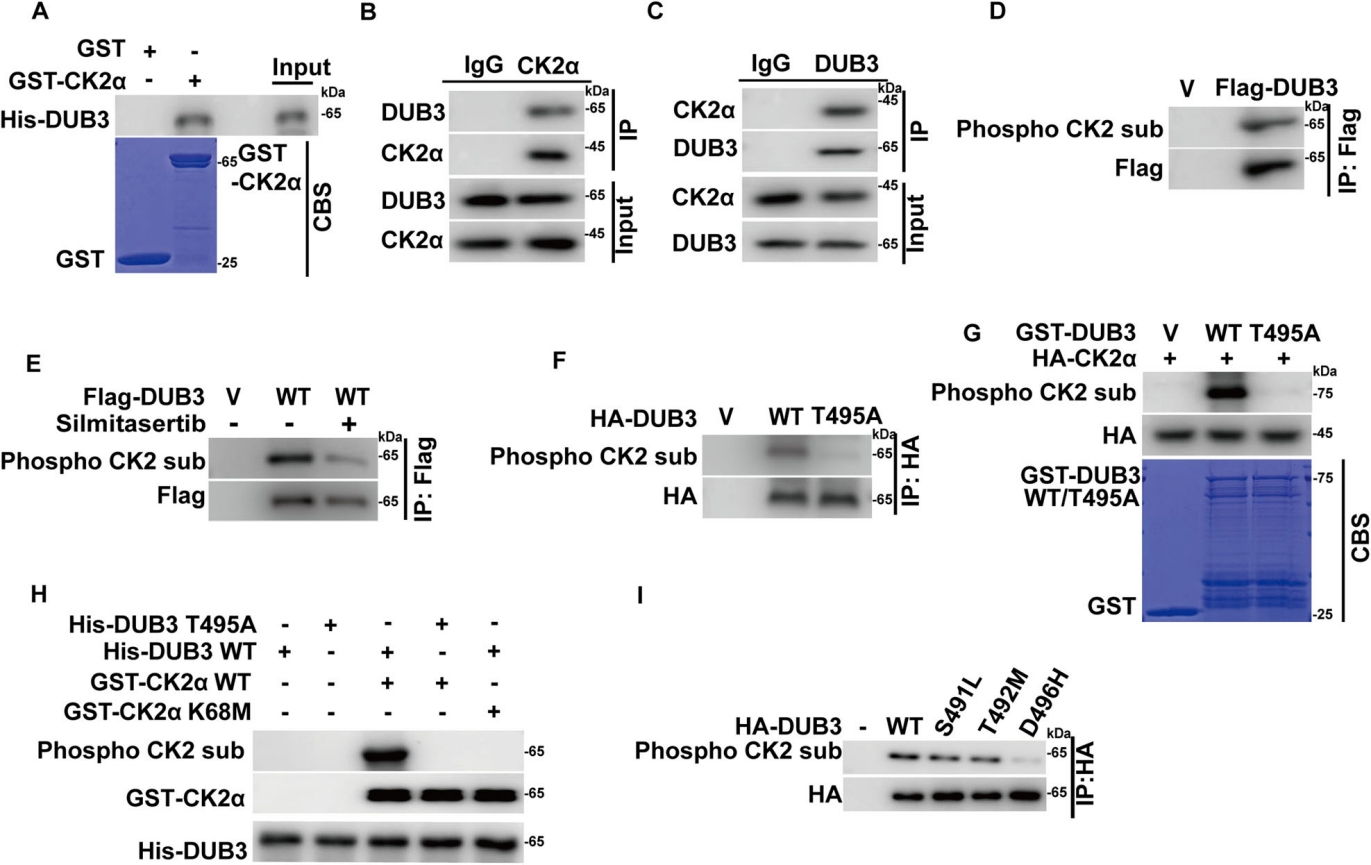
CK2α interacts with DUB3 and phosphorylates DUB3 at Thr495. **A** The direct interaction between bacterial purified GST-CK2α and His-DUB3 was measured by an in vitro interaction assay. CBS was performed to examine GST or GST-fusion protein levels. Cell lysates of A2780 and SKOV3 were immunoprecipitated using IgG, anti-CK2α (**B**), or anti-DUB3 (**C**) antibodies. The endogenous interaction of CK2α-DUB3 was measured by immunoblotting. **D** Vector (V) or Flag-DUB3 were transfected into A2780 cells. Cell lysates were immunoprecipitated using anti-Flag affinity gel, and the phosphorylation of DUB3 was examined by phospho-CK2 substrate antibody. **E** Indicated plasmids were transfected into cells and treated with or without Silmitasertib for 2 h. Anti-Flag affinity gel was used to immunoprecipitate Flag-DUB3, and a phospho-CK2 substrate antibody was used to measure DUB3 phosphorylation by immunoblotting. **F** Indicated constructs were transfected into cells. HA-protein agarose was used to immunoprecipitate DUB3, and a phospho-CK2 substrate antibody was used to measure the phosphorylation of DUB3. **G** Bacterial purified GST, GST-DUB3 or GST-DUB3 T495A were incubated with immunoprecipitated HA-CK2α by HA-protein agarose in the kinase reaction buffer. A Phospho-CK2 substrate antibody was used to measure the phosphorylation of DUB3. CBS was performed to examine GST and GST-fusion protein levels. **H** In vitro kinase assay was performed by mixing purified WT GST-CK2α or GST-CK2α K68M proteins with purified His-DUB3 WT or His-DUB3 T495A proteins. A phospho-CK2 substrate antibody was used to detect the phosphorylation levels of DUB3. **I** Indicated constructs were transfected into cells. HA-protein agarose was used to immunoprecipitate DUB3, and a phospho-CK2 substrate antibody was used to measure the phosphorylation of DUB3.

Since both CK2α and DUB3 inhibit the ubiquitination and degradation of YAP1, we speculated that DUB3 might act as the potential linker between CK2α and YAP1. As shown in Fig. 5A, the interaction between purified GST-CK2α and His-DUB3 was clearly observed in vitro. This endogenous interaction was also validated in A2780 cells (Fig. 5B, C). In addition, by using a phospho-CK2 substrate antibody, the phosphorylation of DUB3 was observed which was significantly mitigated by pretreatment of CK2 inhibitor Silmitasertib (Fig. 5D, E). We next analyze the amino acid sequence of DUB3 and found that only the Thr495 consensus sequence of DUB3 could fully match to the CK2α recognition motif(pS/T-D/E-X-D/E) [35]. And the phosphorylation of DUB3 by using phospho-CK2 substrate antibody was completely abrogated by the T495A mutant (Fig. 5F). The results of in vitro kinase assay also showed that active CK2α phosphorylated GST-DUB3 WT but not the T495A mutant (Fig. 5G, H). These data validated that Thr495 is the primary CK2α-mediated phosphorylation site in DUB3.

Through analyzing the public database (cBioPortal database), we found that several DUB3 mutations including S491L, T492M and D496H were located within the consensus sequence motif of CK2α substrates in certain types of cancers. We then investigated whether these cancer-related DUB3 mutations could be phosphorylated by CK2α. As shown in Fig. 5I, the DUB3 D496H mutant but not the S491L and T492M mutants was defective in such phosphorylation, supporting the importance of the acidic residue at position +1 (pS/T-D/E-X-D/E) for the substrate recognition and phosphorylation by CK2 [35].

### CK2α-mediated phosphorylation of DUB3 promotes YAP1 stability and oncogenic function

**Fig. 6:**
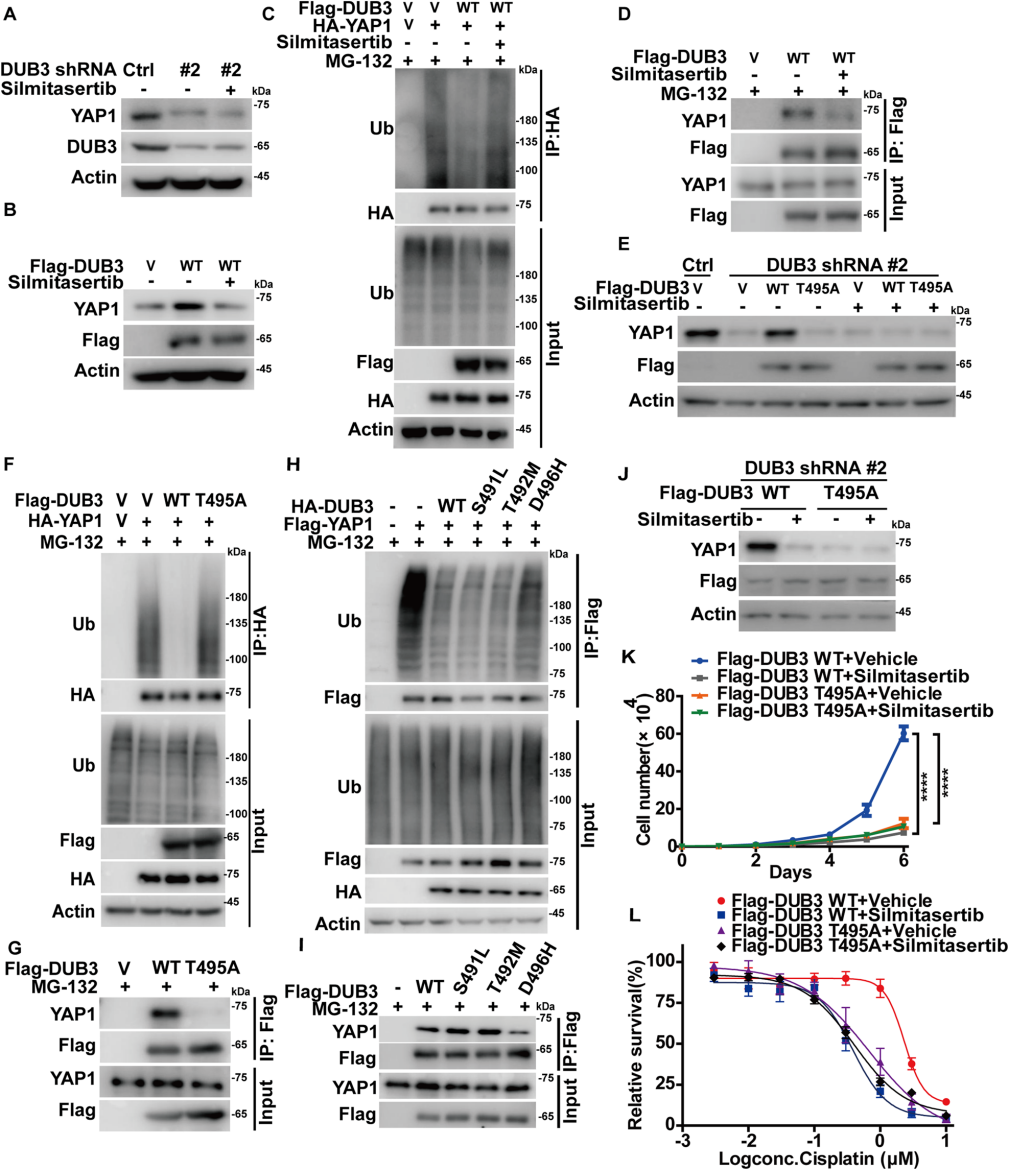
CK2α-mediated phosphorylation of DUB3 promotes YAP1 stability and oncogenic function. **A** Control (Ctrl) or DUB3-depleted A2780 cells were subjected to DMSO or Silmitasertib for 24 h. Protein levels of YAP1 and DUB3 were examined by immunoblotting. **B** Vector (V) or Flag-DUB3 was transfected into A2780 cells and then subjected to DMSO or Silmitasertib for 24 h. The protein level of YAP1 was examined by immunoblotting. **C** Indicated constructs were transfected into cells followed by the treatment with DMSO or Silmitasertib for 24 h. MG-132 (10 μM) was then administered for 10 h. HA-protein agarose was used to immunoprecipitate YAP1, and immunoblotting was performed to measure the polyubiquitylated YAP1. **D** Vector (V) or Flag-DUB3 WT was transfected into cells and then treated with DMSO or Silmitasertib. MG-132 (10 μM) was then administered for 10 h. Anti-Flag affinity gel was used to immunoprecipitate Flag-DUB3 and the interaction between DUB3 and YAP1 was measured by immunoblotting. **E** Indicated constructs were transfected into Control (Ctrl) or DUB3-depleted A2780 cells. Cells were subjected to the treatment with DMSO or Silmitasertib. The protein level of YAP1 was examined by immunoblotting. **F** Indicated constructs were transfected into cells, and MG-132 (10 μM) was then administered for 10 h. HA-protein agarose was used to immunoprecipitate YAP1, and immunoblotting was performed to measure the polyubiquitylated YAP1. **G** Indicated constructs were transfected into cells. Anti-Flag affinity gel was used to immunoprecipitate Flag-DUB3, and the interaction of DUB3-YAP1 was measured by immunoblotting. **H** Indicated constructs were co-transfected into cells followed by treatment of MG-132 (10 μM) for 10 h. Anti-Flag affinity gel was used to immunoprecipitate YAP1, and immunoblotting was used to measure the ubiquitination of YAP1. **I** Indicated constructs were transfected into cells. Anti-Flag affinity gel was used to immunoprecipitate Flag-DUB3, and the interaction of DUB3-YAP1 was measured by immunoblotting. **J** Indicated constructed were transfected into DUB3-depleted A2780 cells and then subjected to the treatment with Silmitasertib. The protein level of YAP1 was examined by immunoblotting. Cell proliferation (**K**) and survival at indicated concentrations of Cisplatin were measured (**L**).

The role of CK2α-mediated DUB3 phosphorylation on YAP1 stability and YAP1-dependent malignant processes was next investigated. The treatment of Silmitasertib efficiently reduced the protein level of YAP1 in control cells but failed to cause additional decrease of YAP1 protein level in DUB3-deficient cells (Fig. 6A). Overexpression of DUB3 WT significantly reduced the ubiquitination level of YAP1 and consequently stabilized YAP1, which were markedly mitigated by the pretreatment with Silmitasertib (Fig. 6B, C). These effects of CK2 inhibition on YAP1 might be due to the impaired interaction of DUB3 with YAP1 (Fig. 6D). Next, we determined whether the regulation of YAP1 by CK2α depended on the phosphorylation status of DUB3 at T495. As shown in Fig. 6E, the reconstitution of DUB3 WT dramatically restored YAP1 protein level in endogenous DUB3-deficient cells, meanwhile the treatment of Silmitasertib could markedly mitigated such an effect. On the contrary, the DUB3 T495A mutant failed to rescue the decreased YAP1 level caused by the depletion of DUB3, which might be due to the impaired interaction of DUB3 T495A with YAP1 and the subsequent inability to deubiquitinate YAP1 (Fig. 6F, G). We also investigated the effect of DUB3 cancer-related mutations on the ubiquitination and turnover of YAP1. Compared to DUB3 WT, S491L and T492M mutants, the phosphorylation-defective mutant DUB3 D496H exhibited much weaker binding affinity to YAP1 as well as impaired ability to cleave the poly-ubiquitination of YAP1 (Fig. 6H-I). These results indicate the critical role of CK2α-mediated phosphorylation of DUB3 on the deubiquitination and stabilization of YAP1.

The functional consequence of CK2α-mediated DUB3 phosphorylation was further examined. As shown in Fig. 6J–L and Fig. S5A–C, reconstitution of DUB3 WT in endogenous DUB3-deficient cells markedly increased cell proliferation and desensitized cancer cells to cisplatin, whereas reconstitution of the T495A mutant failed to affect YAP1 protein level and displayed a strong suppressive effect on ovarian cancer cells. Consistently, the treatment with Silmitasertib markedly suppressed the proliferation and resistance to cisplatin in cells reconstituted with DUB3 WT, but had no obvious effect in cells reconstituted with T495A. Taken together, these results indicate that the phosphorylation of DUB3 at T495 by CK2α is pivotal for promoting tumor progression through stabilizing YAP1.

### Upregulated expressions of CK2α and DUB3 are positively correlated with YAP1 expression in ovarian cancer

**Fig. 7:**
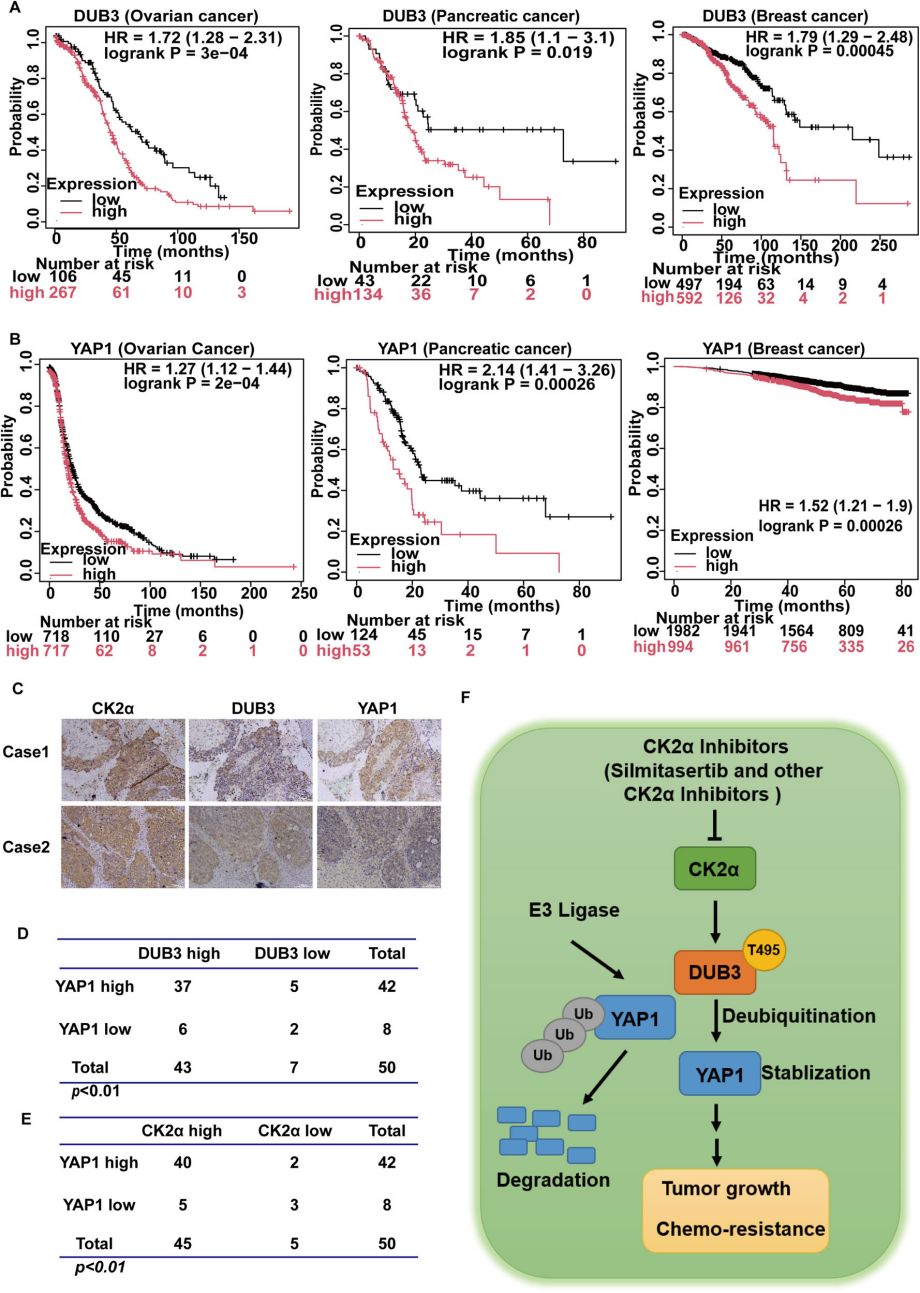
Upregulated expressions of CK2α and DUB3 positively correlate YAP1 expression in ovarian cancer. **A** Survival curve evaluating the prognostic value of DUB3 in ovarian cancer, pancreatic cancer and breast cancer patients were generated using the Kaplan-Meier plotter. **B** Survival curve evaluating the prognostic value of YAP1 in ovarian cancer, pancreatic cancer and breast cancer patients were generated by the Kaplan-Meier plotter. **C** Immunohistochemistry was performed to examine CK2α, DUB3, and YAP1 expressions in ovarian cancer specimens. The expression of YAP1 is positively correlated to DUB3 (**D**) or CK2α (**E**) expression in ovarian cancer samples. **F** Schematic illustration of the finding that CK2α-mediated phosphorylation is crucial for the deubiquitination process of YAP1 by DUB3, thereby promoting cancer progression by stabilizing YAP1.

Through the analysis of public gene expression databases, high levels of DUB3 and YAP1 were found to correlate with poor relapse-free survival in ovarian, pancreatic, and breast cancer (Fig. 7A, B). We next investigated the clinical relevance of CK2α-DUB3-YAP1 axis in ovarian cancer specimens using immunohistochemistry analysis. The upregulated expression of DUB3 positively correlated with YAP1 in ovarian cancer specimens (Fig. 7C, D). Specifically, 37 cancer tissues with high YAP1 levels also exhibited relative high intensity of DUB3 staining, whereas 25% of samples with lower YAP1 levels displayed lower DUB3 staining. Additionally, we observed that YAP1 staining level positively correlated with CK2α expression. Approximately 95.24% of samples with high YAP1 levels exhibited high CK2α expression, while 37.5% of samples with low YAP1 levels were associated with less CK2α expression (Fig. 7C, E). Collectively, these results demonstrate that the dysregulated CK2α-DUB3-YAP1 axis contributes to the tumor progression of ovarian cancer and the therapeutic strategies targeting this axis could be of benefit in the clinical management of ovarian cancer.

## Discussion

Here, we uncover several unexpected findings with critical clinical implications. First, we identify the CK2α-DUB3 axis as an uncharacterized upstream signaling pathway regulating cellular YAP1 abundance in multiple cancer types including ovarian cancer, which critically contributes to tumor progression and chemotherapy resistance. Second, we mechanistically demonstrate that CK2α-catalyzed phosphorylation of DUB3 at T495 is a novel and common post-translational modification ensuring the oncogenic maintenance of YAP1 protein stability in multiple malignant cancers. Furthermore, we provide strong evidence that targeting the CK2α-DUB3 axis could be effective in suppressing tumor progression in ovarian cancer and other cancers harboring aberrantly upregulated YAP1 (Fig. 7F).

YAP1 has been implicated in tumor progression of a variety of human cancers, which renders it an appealing therapeutic target. However, due to the lack of accessible binding pockets in YAP1 for small molecule compounds, drug development to direct target YAP1 is yet not successful for the treatment of human cancer. Thus, developing alternative strategies to destabilize YAP1 might improve the clinical outcome of lethal cancers harboring aberrantly upregulated YAP1. CK2 is a ubiquitously expressed and constitutively active serine/threonine kinase in eukaryotes, which is composed of two catalytic subunits and two regulatory subunits. CK2 has been demonstrated to phosphorylate the Ser or Thr residue of acidic motif (pS/p-D/E-X-D/E) in substrates and critically regulate the normal embryonic development, cell survival, DNA damage response and circadian rhythms regulation [36–39]. Although an aberrantly elevated CK2 catalytic subunit is commonly detected and associated with poor prognosis in multiple cancers [40–42], its mechanism of action remains elusive in ovarian cancer. Here, we reveal a novel functional role of CK2 to stabilize YAP1 in ovarian cancer and other types of cancer. Firstly, we screened out that the CK2 inhibitor Silmitasertib as well as the genetic ablation of CK2α could significantly decrease the YAP1 protein levels (Fig. 1B, C, Fig. 2A, B). Secondly, such an effect of CK2 on YAP1 is mediated by ubiquitin-proteasome-dependent degradation, which is evidenced by the increased ubiquitination and shortened half-life of YAP1 in CK2α-depleted cells or in cells treated with Silmitasertib (Fig. 2C–H, Fig. S3A–D). More importantly, the tight correlation between CK2α and YAP1 was observed in ovarian cancer specimens (Fig. 7). Lastly, targeting CK2α could markedly suppress cancer cell proliferation and sensitize cancer cells to chemotherapeutic drugs both in vitro and in vivo (Fig. 1E–L, Fig. S2C–N). The suppressive effects of targeting CK2 are largely YAP1-dependent since the reconstitution of YAP1 markedly rescues these suppressive phenotypes induced by targeting CK2α (Fig. 1E–L, Fig. S2C–N). However, it is also possible that additional mechanisms might be engaged in CK2α-promoted ovarian cancer progression as the reconstitution of YAP1 could not completely restored phenotypical changes caused by targeting CK2α in ovarian cancer cells (Fig. 1E–L, Fig. S2C–N). In glioblastoma, CK2 was reported to phosphorylate and stabilize PRMT6, which enhances the methylation of RCC1, thereby facilitating RCC1 localization in chromatin and contributing to tumorigenicity and progression of glioblastoma [29]. CK2-catalyzed phosphorylation is also essential for the interaction of CSN2 and Cullin RING ligase 4 (CRL4), while high glucose level induces O-GlcNAcylation of CK2 and causes the dissociation of CSN-CRL4. The released CRL4 assembles the CRL4-COP1 E3 ligase complex and promotes the p53 degradation in breast cancer [43]. Whether and how these CK2 substrates besides YAP1 are potentially involved in ovarian cancer progression need further investigation.

Emerging evidence demonstrates CK2α directly phosphorylate and regulate the stability, activity or protein-protein interaction of its substrates. CK2α was reported to phosphorylate PML at Ser 517 and promote its ubiquitination-mediated degradation, thereby promoting tumorigenesis and development of lung cancer [44]. In hepatocellular carcinoma, the treatment of insulin-like growth factor 1 promoted CK2-mediated CLOCK S106 phosphorylation, which consequentially disrupts the CLOCK-BMAL1 heterodimer and suppresses the expression of its downstream genes [17]. To our surprise, YAP1 is not the direct substrate of CK2α as neither direct interaction of purified CK2α with YAP1 nor CK2α-mediated phosphorylation of YAP1 was observed (Fig. 3A, B). Instead, our findings reveal the deubiquitinase DUB3 functions as the linking factor between CK2α and YAP1. Our previous study and others demonstrated that DUB3, also known as USP17L2, could stabilize the key EMT-factor Snail1 and induce EMT and promote TNBC metastasis [19, 45]. Here, our study uncovers a novel tumor-promoting function of DUB3 to stabilize YAP1 in ovarian cancer, which is post-translationally controlled by CK2α. Several lines of evidence support are as follows. 1) DUB3 directly interacts with YAP1 and stabilizes YAP1 by catalyzing its deubiquitination process (Fig. 3F, Fig. 4A–H). 2) DUB3 promotes tumor progression of ovarian cancer in a YAP1-dependent manner (Fig. 4I–M, Fig. S4F–H). 3) CK2α directly interacts and phosphorylates DUB3, which markedly enhance the ability of DUB3 to deubiquitinate and stabilize YAP1 (Fig. 5A–D, Fig. 6A–D). 4) The tumor-suppressive effect of CK2α inhibition could only detected in cells reconstituting DUB3 WT, but not the phosphorylation defective mutant T495A (Fig. 6J–L, Fig. S5A–C). These findings strongly support the importance of CK2α-mediated phosphorylation in controlling the stability of YAP1 in ovarian cancer.

CK2 is widely recognized as an acidophilic kinase, preferring phosphorylation sites surrounded by acidic residues, particularly aspartate and glutamate (D/E), at both the N- and C-termini. Emerging evidence suggests that D/E specificity of CK2 is especially strong at substrate positions +3 and +1 (S/T[E/D]x[E/D]), as demonstrated through various modeling approaches [46–48]. The DUB3 D496H mutation occurs at substrate positions +1, potentially disrupting CK2 recognition and leading to impaired phosphorylation of DUB3 (Fig. 5I). Although the D496H mutation occurs at a relatively low frequency compared to other mutations in DUB3, it is associated with certain cancers, including lung adenocarcinomas. This mutation can be notable because it occurs within the CK2 substrate consensus sequence, which may play a crucial role in modulating DUB3 phosphorylation at Thr 495. This phosphorylation is essential for the regulation of DUB3-mediated deubiquitination and stabilization of YAP1, and potentially other oncogenic substrates such as Snail1 in triple-negative breast cancer [19, 45]. Given the well-established role of YAP1 and Snail1 as tumor promoters, their stabilization could contribute to more aggressive tumor characteristics, including an increased cancer stem cell population, enhanced cell proliferation, and metastasis. Moreover, the D496H mutation may influence tumor cell sensitivity to chemotherapeutic agents. Despite the low frequency of D496H mutation, its clinical significance has yet to be fully characterized. Comprehensive genetic profiling that identifies less common mutations like D496H, along with further exploration in its biological significance, is essential for understanding its implication in cancer biology and therapeutic responses. This understanding may ultimately contribute to more personalized treatment strategies and improved outcomes for specific patient populations.

Collectively, we reveal that targeting the CK2α-DUB3 axis might be appealing for suppressing ovarian cancer by destabilizing YAP1. More intriguingly, the stabilization of YAP1 by the CK2α-DUB3 axis could be a common regulatory mechanism in PDAC, CRC, HCC, TNBC and RCC cancer cells (Fig. 1M, N, Fig. 4C). Considering that Silmitasertib was well tolerated and has been approved by FDA for the treatment of cholangiocarcinoma, more preclinical and clinical studies are further needed in these lethal cancers with aberrant upregulation of CK2α and YAP1.

## Supplementary information

Supplementary figure

Supplemental Material-Original Data

## Data Availability

All data generated or analyzed during this study are available within the article and Supplementary Files, or available from the authors upon request.
