# Supplementary material for: CK2α-mediated phosphorylation of DUB3 promotes YAP1 stability and oncogenic functions
Source: Cell Death Dis. 2025 Jan 18;16(1):27. doi: 10.1038/s41419-024-07323-z (PMC11743126; doi:10.1038/s41419-024-07323-z)
Supplement: Supplementary file 1 — Supplementary figure [file 41419_2024_7323_MOESM1_ESM.docx]

**
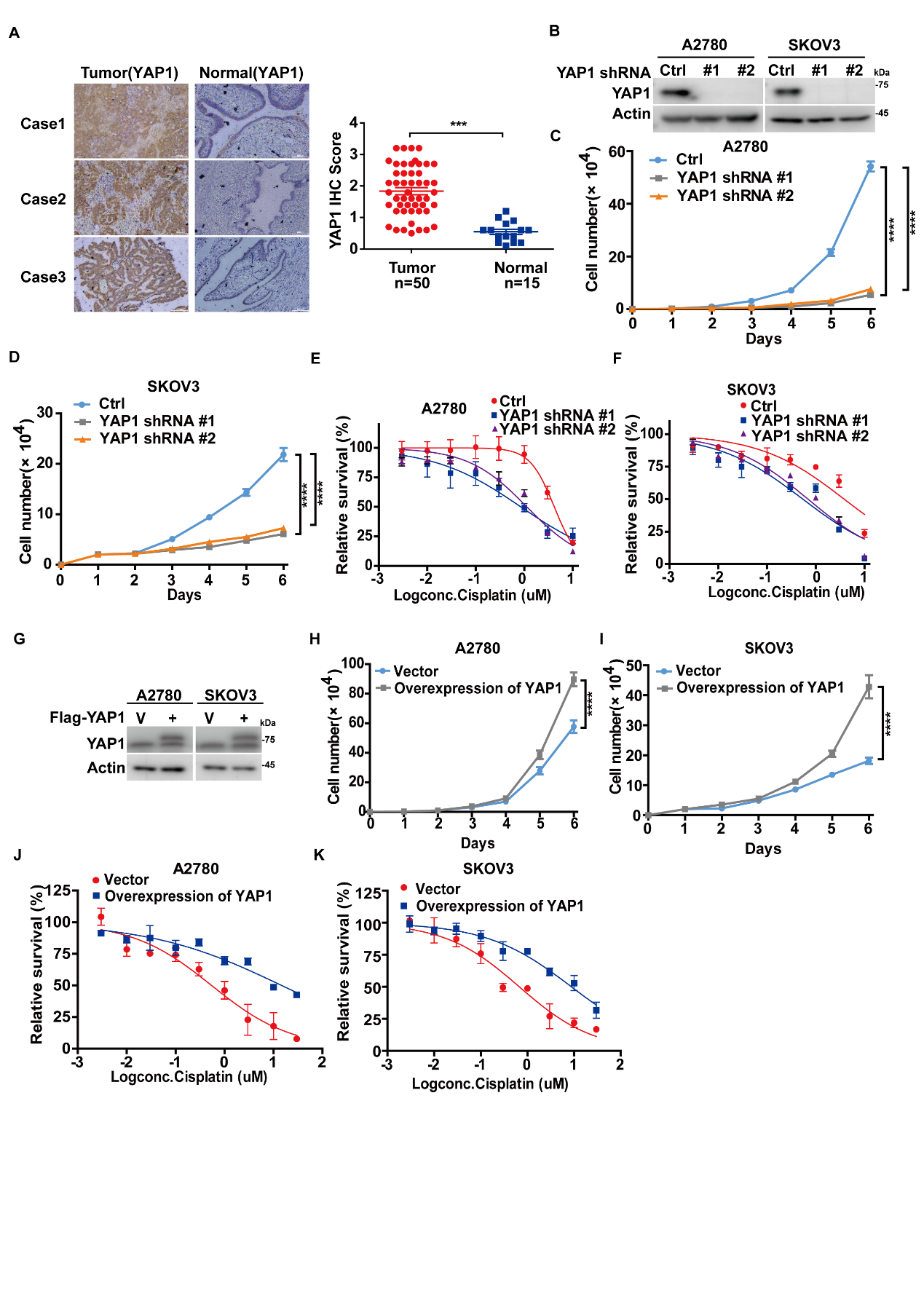
**

**Figure S1. YAP1 is overexpressed in ovarian cancer tissues and the depletion of YAP1 in ovarian cancer cells exhibits suppressive effects. A** Immunohistochemistry was performed to measure YAP1 protein expression in ovarian cancer and normal specimens. Representative images of YAP1 immunohistochemistry (left) and the statistical graph (right) are shown. Scale bar, 100 μm. **B** YAP1 was depleted by its specific shRNAs in A2780 and SKOV3 cells. The protein level of YAP1 was examined by immunoblotting. **C, D** Cell proliferation of (**B**) was examined and analyzed. **E, F** Cells from **(B)** were exposed to indicated concentrations of cisplatin, and CCK8 assay was performed to measure cell survival. **G** Flag-YAP1 was transfected into A2780 cells and SKOV3 cells. The protein level of YAP1 was examined by immunoblotting. **H, I** Cell proliferation of (**G**) was examined and analyzed. **J, K** Cells from (**G**) were exposed to indicated concentrations of cisplatin, and CCK8 assay was performed to measure cell survival.


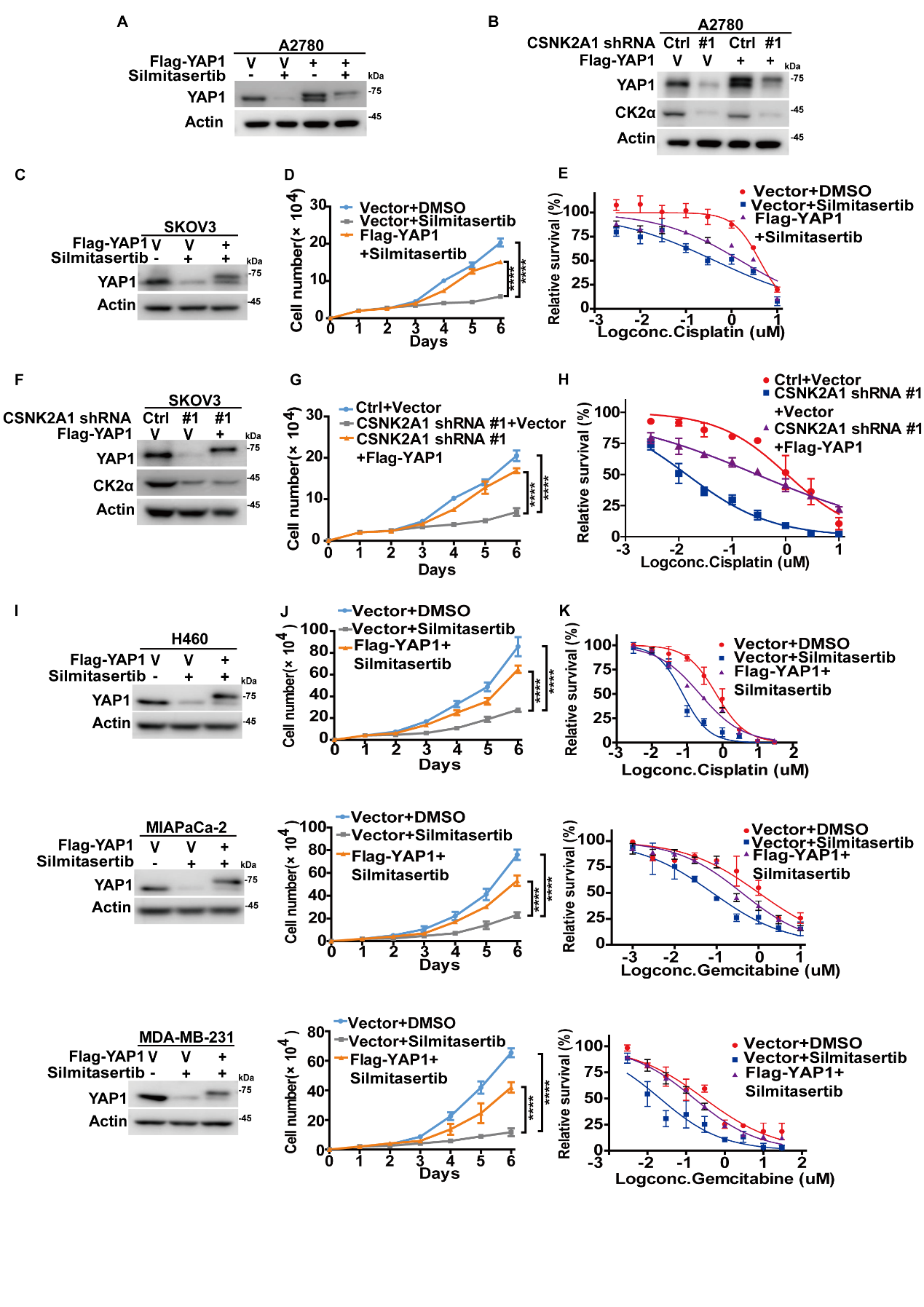


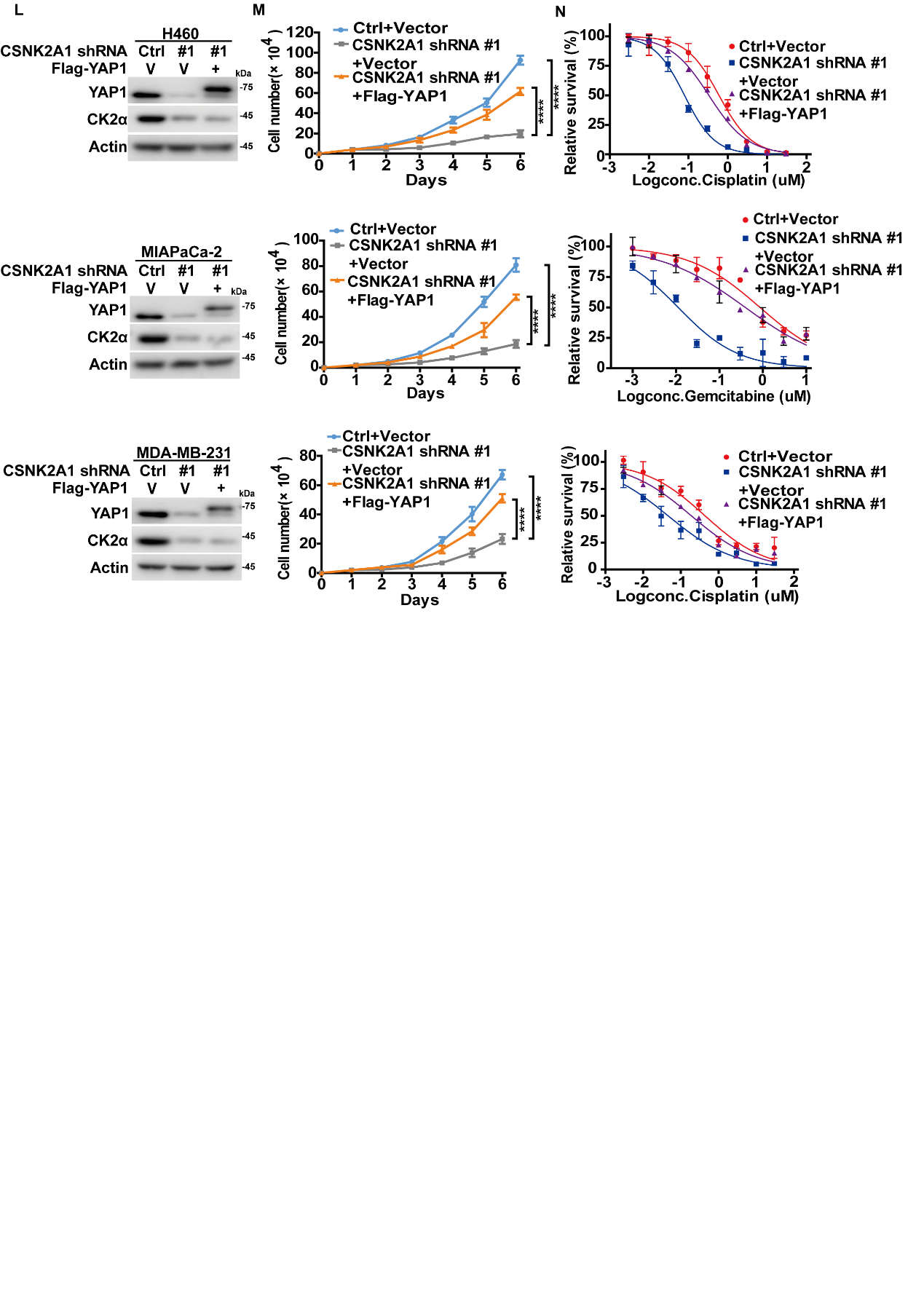


**Figure S2. Protein kinase CK2α regulate YAP1 in a variety of cancers.** **A** A2780 cells were transfected with vector (V) or Flag-YAP1, and then treated with either DMSO or Silmitasertib. And protein levels of YAP1 were examined by immunoblotting. **B** Control (Ctrl) or CK2α-depleted A2780 cells were transfected with vector (V) or Flag-YAP1. The protein levels of YAP1 and CK2α were examined by immunoblotting. **C** Vector (V) or Flag-YAP1 were transfected into SKOV3 cells and then treated with DMSO or Silmitasertib. The protein level of YAP1 was examined by immunoblotting. **D** Cell proliferation of (**C**) was measured and analyzed. **E** Cells from (**C**) were exposed to indicated concentrations of cisplatin, and CCK8 assay was performed to measure cell survival. **F** Vector (V) or Flag-YAP1 were transfected into control (Ctrl) or CK2α-depleted SKOV3 cells, and YAP1 protein level was measured by immunoblotting. **G** Cell proliferation of (**F**) was measured and analyzed. **H** Cells from (**F**) were exposed to indicated concentrations of cisplatin, and CCK8 assay was performed to measure cell survival. **I** Vector (V) or Flag-YAP1 were transfected into H460, MIAPaCa-2, and MDA-MB-231 cells and then treated with DMSO or Silmitasertib. The protein level of YAP1 was examined by immunoblotting. **J** Cell proliferation of (**I**) was measured and analyzed. **K** Cells from (**I**) were exposed to indicated concentrations of cisplatin, and CCK8 assay was performed to measure cell survival. **L** Vector (V) or Flag-YAP1 was transfected in control (Ctrl) or CK2α-depleted H460, MIAPaCa-2, and MDA-MB-231 cells, and YAP1 protein level was measured by immunoblotting. **M** Cell proliferation of (**L**) was measured and analyzed. **N** Cells from (**L**) were exposed to indicated concentrations of cisplatin or gemcitabine and CCK8 assay was performed to measure cell survival.


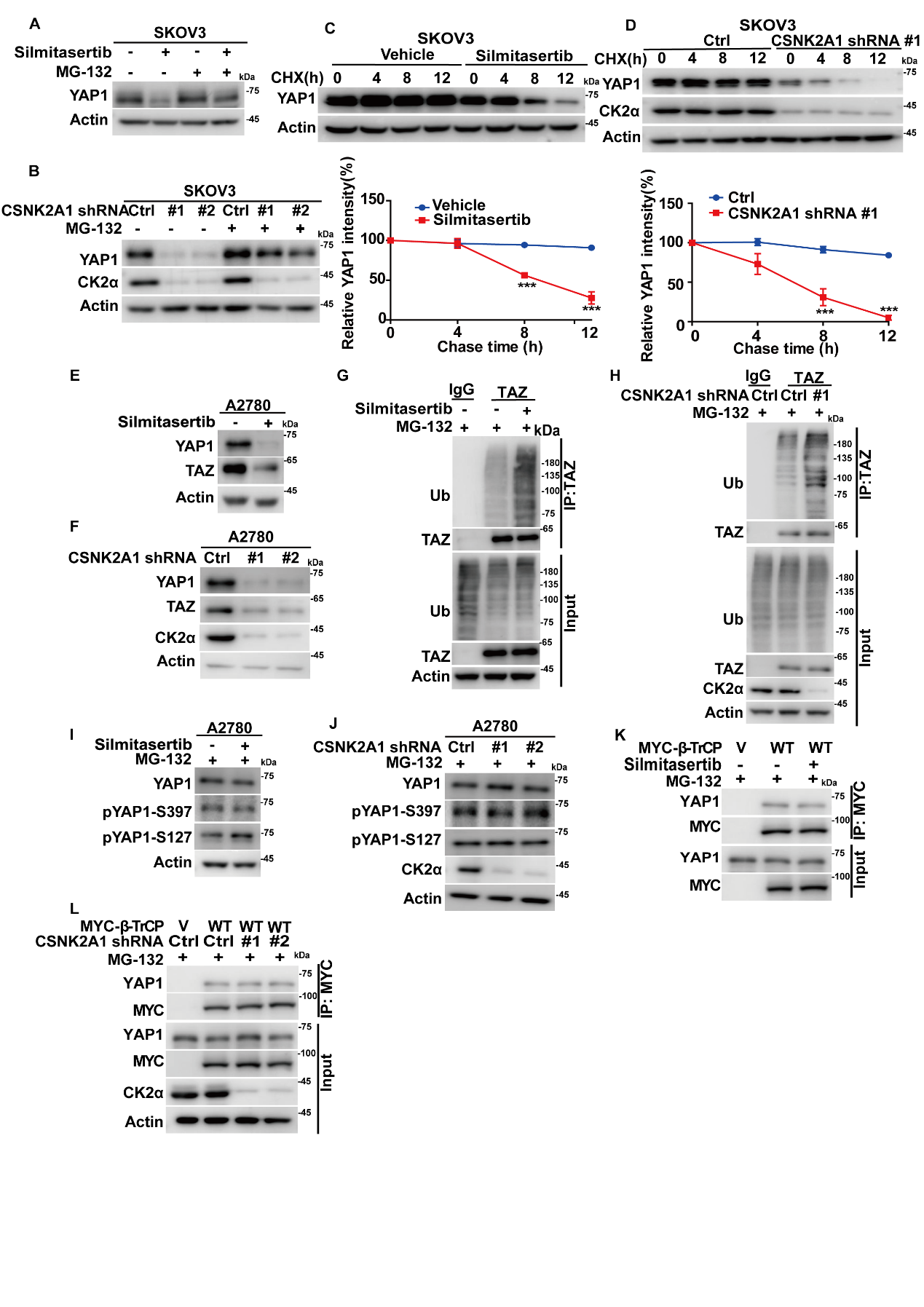


**Figure S3. CK2α stabilizes YAP1.** **A** SKOV3 cells were exposed to DMSO or Silmitasertib followed by the treatment of either DMSO or MG-132 (10 μM) for 10 hours. The protein level of YAP1 was examined by immunoblotting. **B** Control or CK2α-depleted SKOV3 cells were subjected to DMSO or MG-132 (10 μM) treatment. The protein levels of YAP1 and CK2α were examined by immunoblotting. **C** SKOV3 cells were pretreated with DMSO or Silmitasertib followed by cycloheximide (200 μg/mL) treatment. Cell lysate was collected at the indicated times (n=3). The protein level of YAP1 was examined by immunoblotting. The relative level of YAP1 to Actin was analyzed by image J. **D** Control or CK2α-depleted SKOV3 cells were treated with cycloheximide (200 μg/mL). Cell lysate was collected at the indicated times and the protein level of YAP1 was examined by immunoblotting. (n=3). The relative level of YAP1 to Actin was analyzed by image J. **E** A2780 cells were treated with DMSO or Silmitasertib, and western blotting was performed with indicated antibodies. **F** CK2α-depleted A2780 cells were generated. Protein levels of YAP1, TAZ, and CK2α were measured by immunoblotting. **G** Cells were pretreated with DMSO or Silmitasertib followed by 10 hours of exposure to MG-132 (10 μM). Cell lysates were subjected to immunoprecipitation with IgG or anti-TAZ, and the ubiquitination level of TAZ was measured by immunoblotting. **H** Control (Ctrl) or CK2α-depleted cells were treated with MG-132 (10 μM), and cell lysates were subjected to immunoprecipitation with IgG or anti-TAZ. The ubiquitination level of TAZ was measured by immunoblotting. **I** A2780 cells were treated with DMSO or Silmitasertib followed by the treatment of MG-132 (10 μM) for 10 hours. Western blotting was performed with indicated antibodies. **J** Control (Ctrl) or CK2α-depleted A2780 cells cells were treated with MG-132 (10 μM) for 10 hours. Western blotting was performed with indicated antibodies. **K** Cells were transfected with Vector (V) or MYC-β-TRCP and then treated with DMSO or Silmitasertib. MG-132 (10 μM) was then administered for 10 hours. Anti-Myc Magnetic Beads was used to immunoprecipitate MYC-β-TRCP and the interaction between β-TRCP and YAP1 was measured by immunoblotting. **L** Control (Ctrl) or CK2α-depleted cells were transfected with Vector or MYC-β-TRCP and then treated with MG-132 (10 μM) for 10 hours. Anti-Myc Magnetic Beads was used to immunoprecipitate MYC-β-TRCP and the interaction between β-TRCP and YAP1 was measured by immunoblotting.


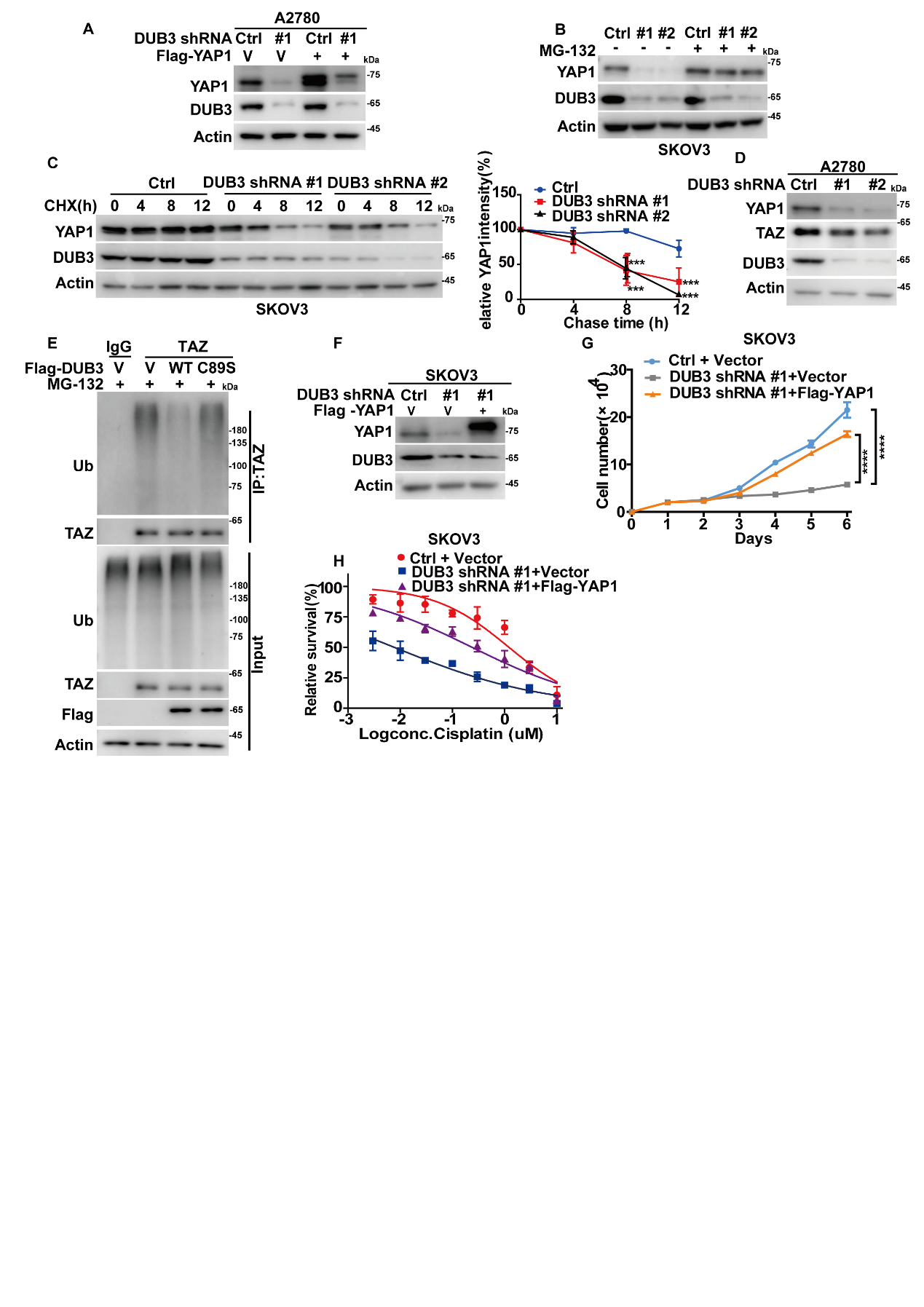


**Figure S4. DUB3 stabilizes YAP1 and promotes the malignant phenotypes of ovarian cancer. A** Control (Ctrl) or DUB3-depleted A2780 cells were transfected with vector (V) or Flag-YAP1. The protein levels of YAP1 and DUB3 were examined by immunoblotting. **B** Control (Ctrl) or DUB3-depleted SKOV3 cells were subjected to DMSO or MG-132 (10 μM) for 10 hours. The protein levels of YAP1 and DUB3 were examined by immunoblotting. **C** Control or DUB3-depleted SKOV3 cells were treated with cycloheximide (200 μg/mL). Cell lysate was collected at the indicated times and the protein level of YAP1 was examined by immunoblotting (n=3). The relative level of YAP1 to Actin was analyzed by image J. **D** Control (Ctrl) or DUB3-depleted A2780 cells were generated, and the protein levels of YAP1, TAZ and DUB3 were measured by immunoblotting. **E** Cells were transfected with indicated constructs, then treated with to MG-132 (10 μM) for 10 hours. Cell lysates were subjected to immunoprecipitation with IgG or anti-TAZ, and the ubiquitination level of TAZ was measured by immunoblotting. **F** Vector (V) or Flag-YAP1 were transfected into control (Ctrl) or DUB3-depleted SKOV3 cells, and protein levels of YAP1 and DUB3 were examined by immunoblotting. **G** The proliferation of cells in (**F**) was measured and analyzed. **H** Cells from (**F**) were exposed to indicated concentrations of cisplatin, and CCK8 assay was performed to measure cell survival.


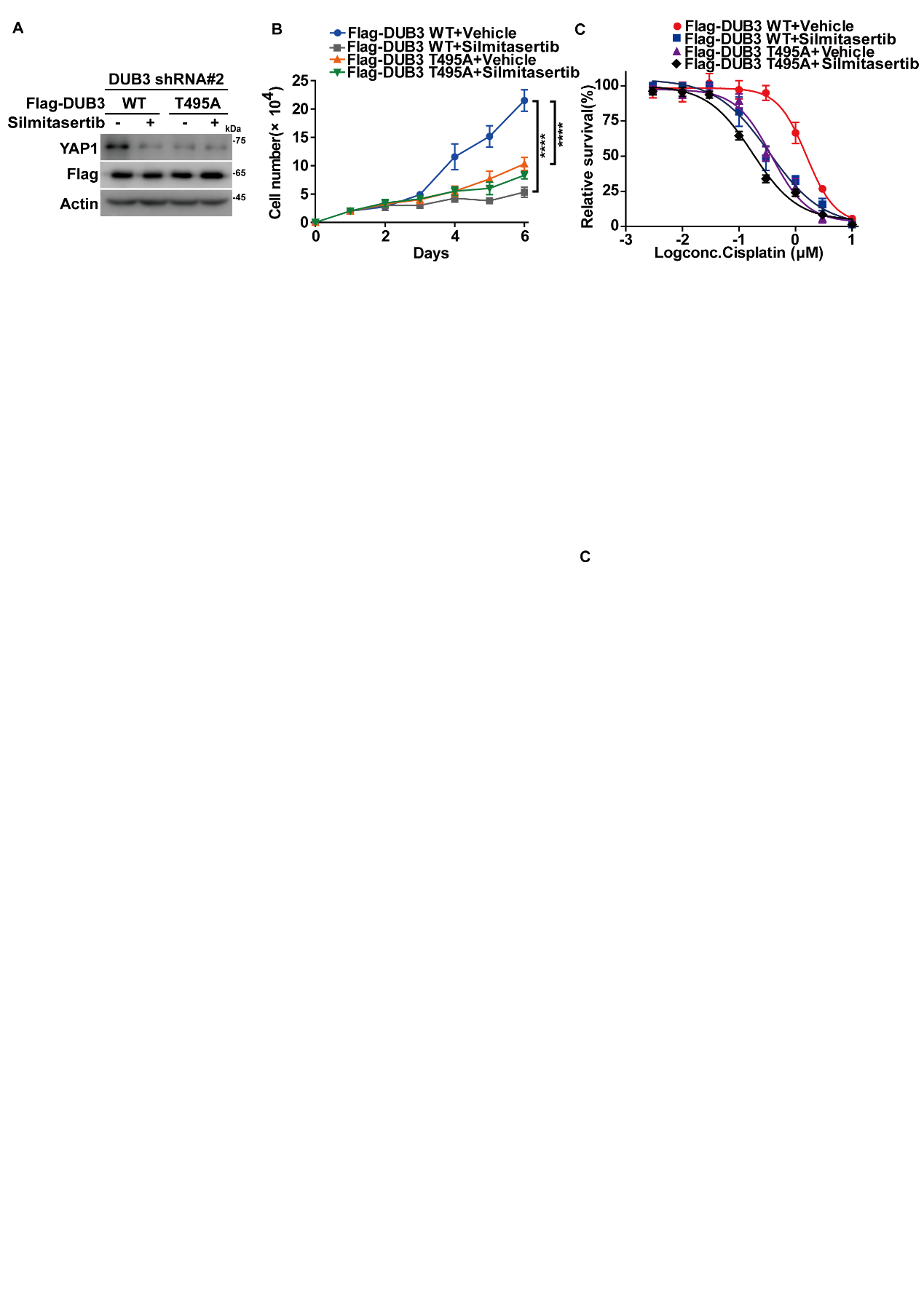


**Figure S5. CK2α-mediated phosphorylation of DUB3 regulates YAP1 protein level and tumor-promoting function.** **A** Flag-DUB3 WT and the T495 mutant were reconstituted into DUB3-depleted SKOV3 cells and then subjected to the treatment of Silmitasertib. The protein level of YAP1 was examined by immunoblotting. **B** The proliferation of cells in (**A**) was measured and analyzed. **C** Cells in (**A**) were exposed to indicated concentrations of cisplatin, and CCK8 assay was performed to measure cell survival.
