# Supplementary material for: CK2α-mediated phosphorylation of DUB3 promotes YAP1 stability and oncogenic functions
Source: Cell Death Dis. 2025 Jan 18;16(1):27. doi: 10.1038/s41419-024-07323-z (PMC11743126; doi:10.1038/s41419-024-07323-z)
Supplement: Supplementary file 2 — Supplemental Material-Original Data [file 41419_2024_7323_MOESM2_ESM.docx]

CK2α-mediated Phosphorylation of DUB3 Promotes YAP1 Stability and Oncogenic Functions

Figure S6.


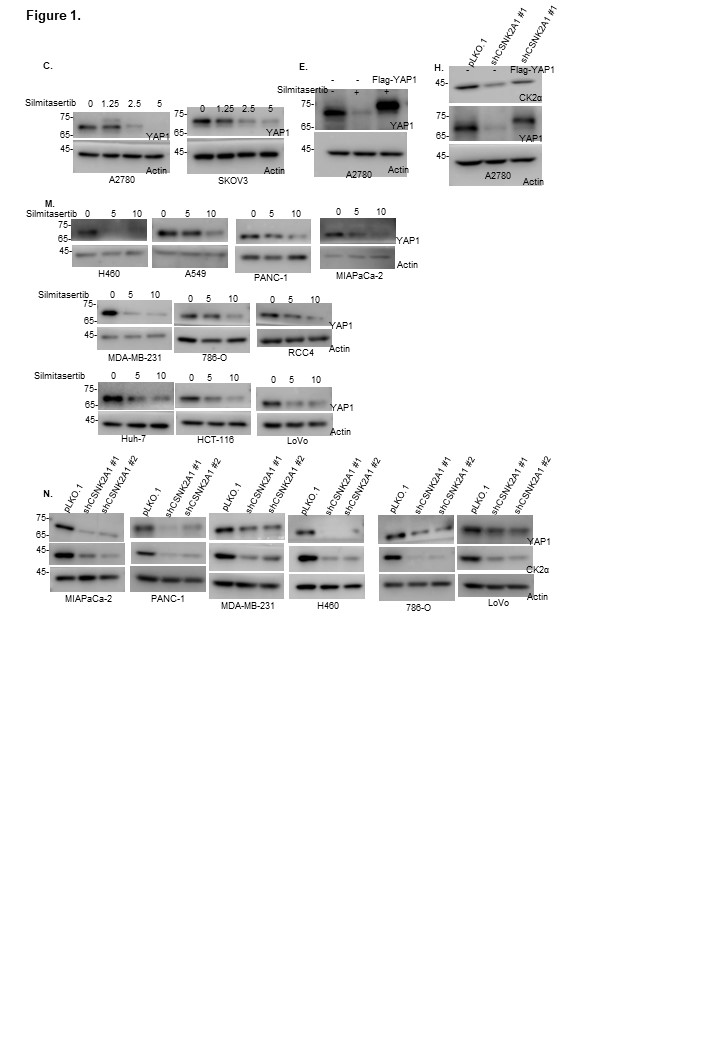


Figure S6: Original scan of the blots presented in the main text. Related to Figure 1.

Figure S7.


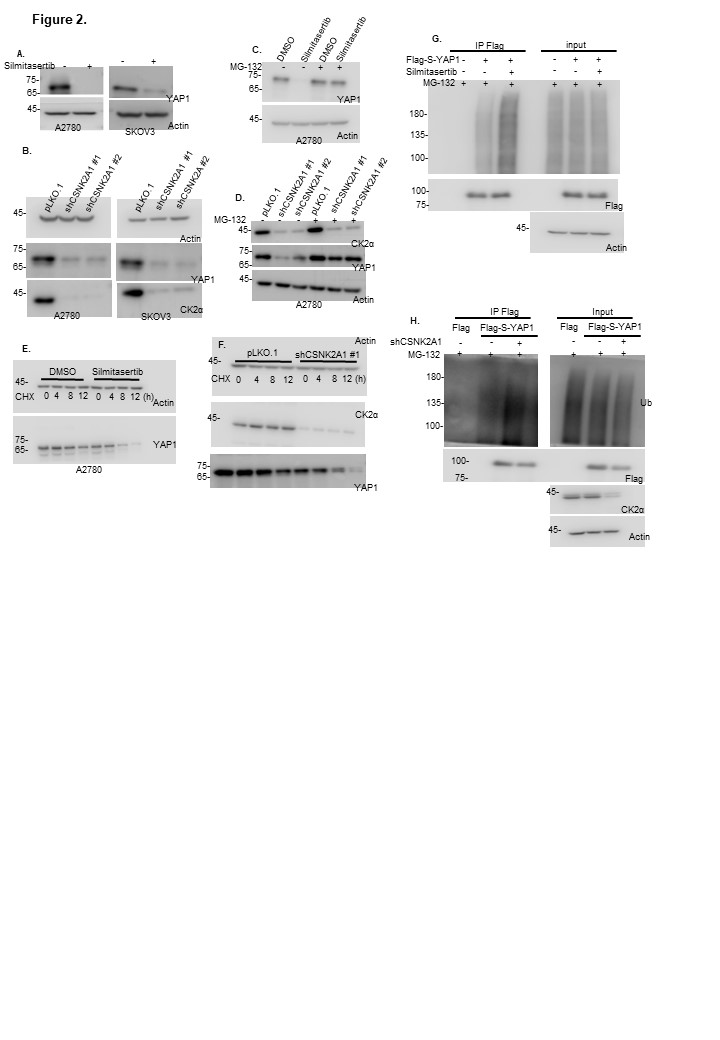


Figure S7: Original scan of the blots presented in the main text. Related to Figure 2

Figure S8.


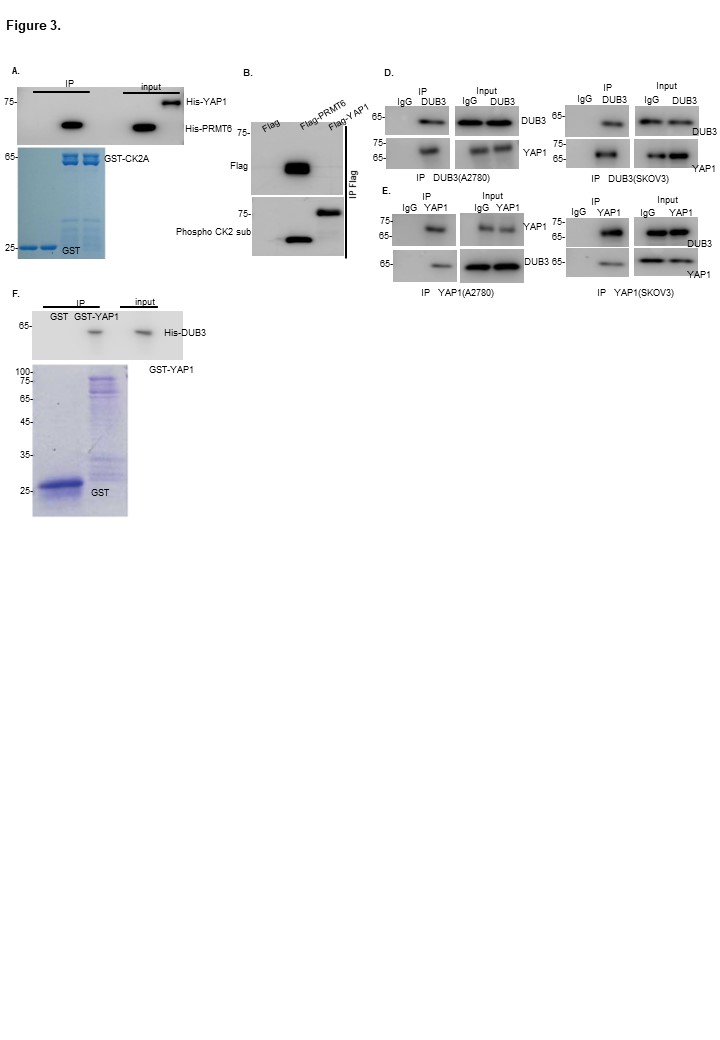


Figure S8: Original scan of the blots presented in the main text. Related to Figure 3

Figure S9


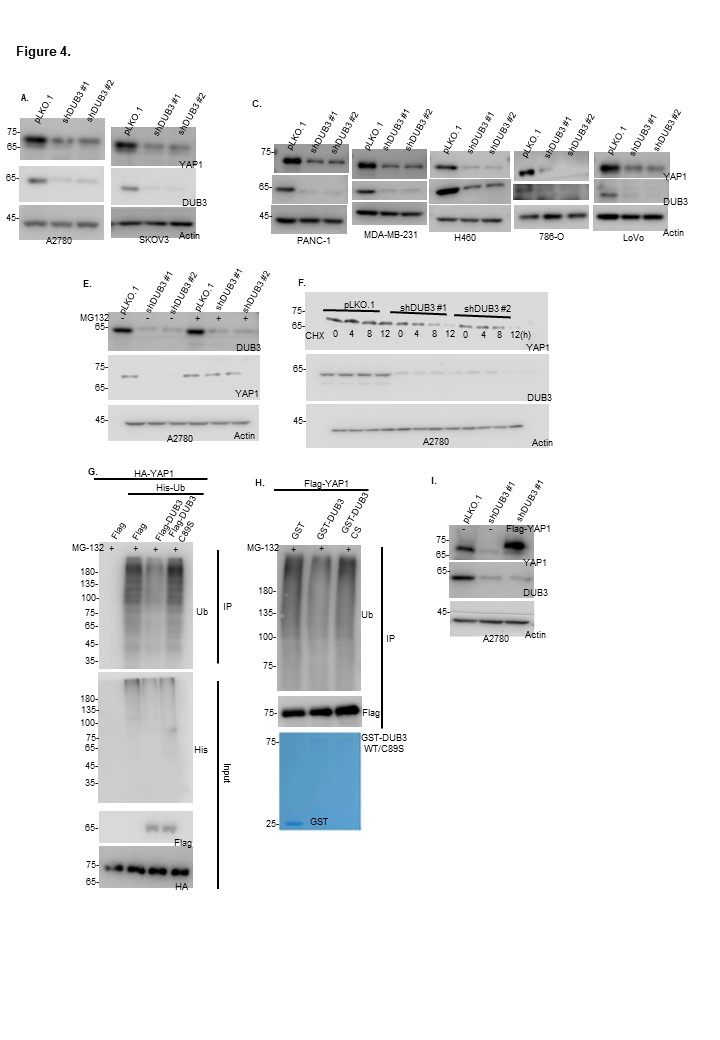


Figure S9: Original scan of the blots presented in the main text. Related to Figure 4

Figure S10


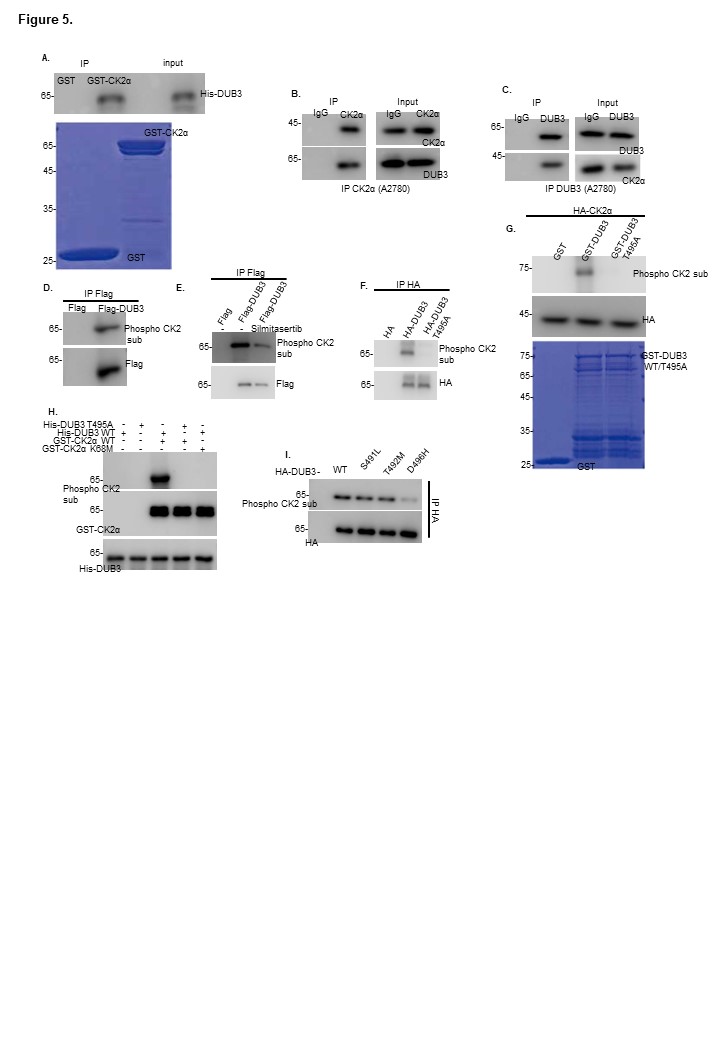


Figure S10: Original scan of the blots presented in the main text. Related to Figure 5

Figure S11


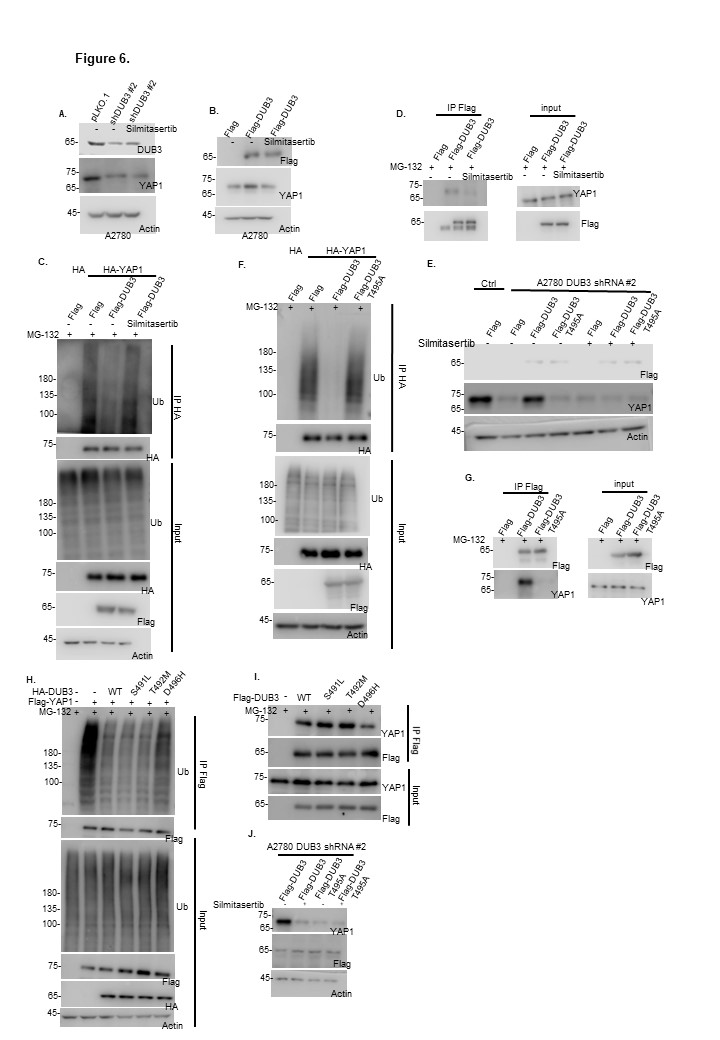


Figure S11: Original scan of the blots presented in the main text. Related to Figure 6

Figure S12


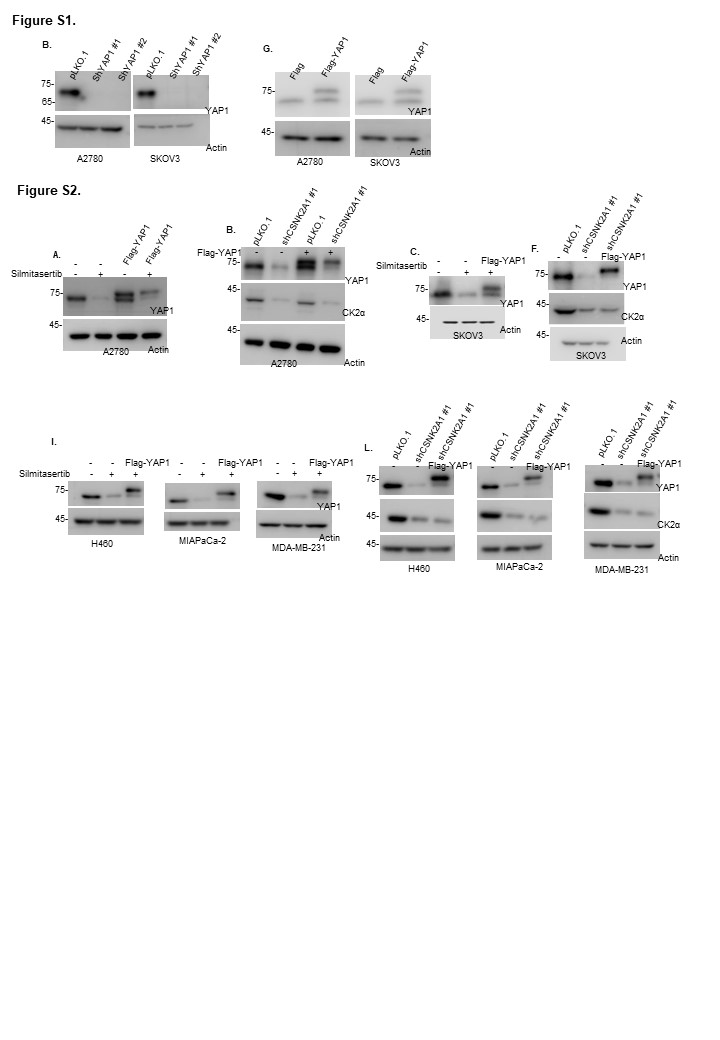


Figure S12: Original scan of the blots presented in the main text. Related to Figure S1 and Figure S2

Figure S13


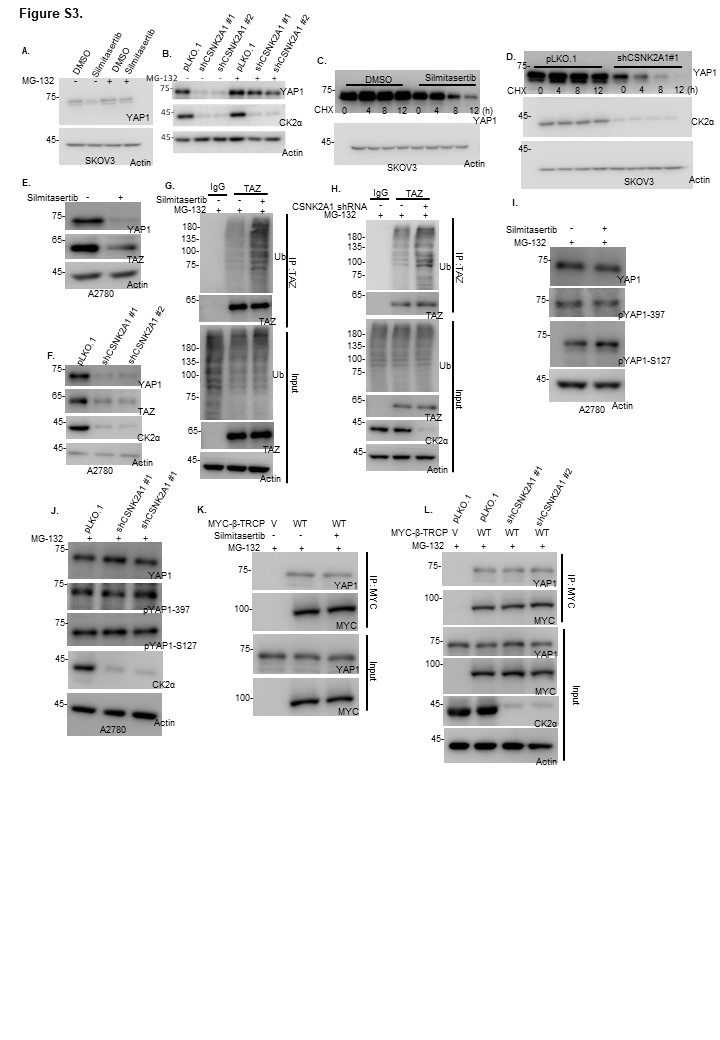


Figure S13: Original scan of the blots presented in the main text. Related to Figure S3

Figure S14


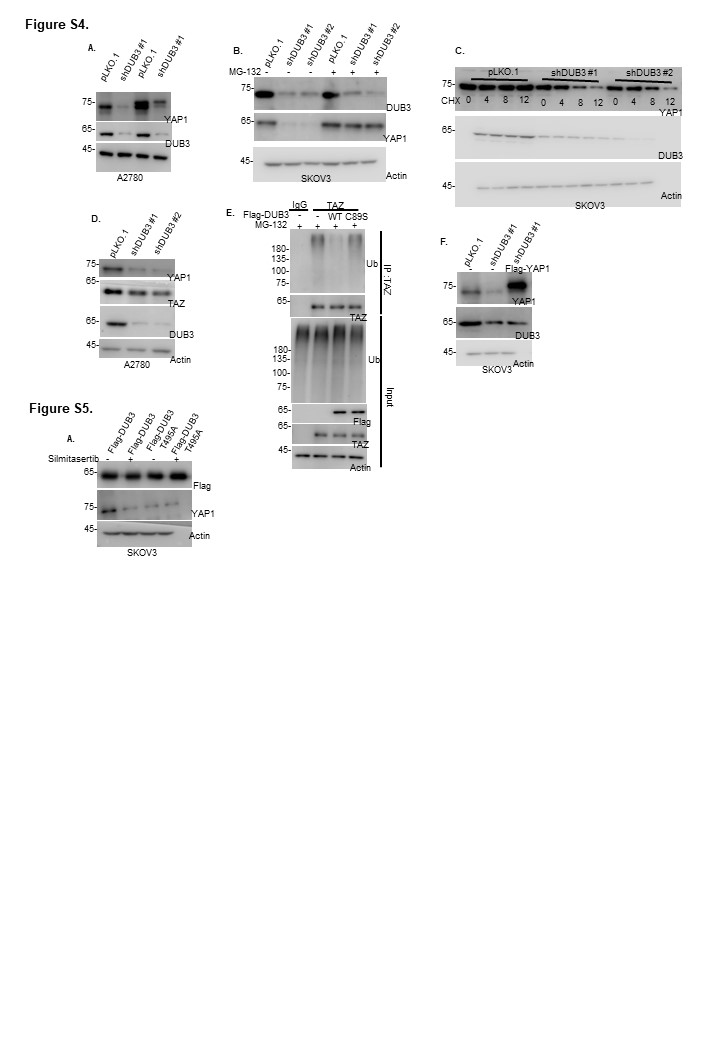


Figure S14: Original scan of the blots presented in the main text. Related to Figure S4 and Figure S5
